# Cooperative Self‐Assembly in Linear Chains Based on Halogen Bonds

**DOI:** 10.1002/cplu.202100093

**Published:** 2021-05-06

**Authors:** Pascal Vermeeren, Lando P. Wolters, Gábor Paragi, Célia Fonseca Guerra

**Affiliations:** ^1^ Department of Theoretical Chemistry, Amsterdam Institute of Molecular and Life Sciences (AIMMS) Amsterdam Center for Multiscale Modeling (ACMM) Vrije Universiteit Amsterdam De Boelelaan 1083 1081 HV Amsterdam The Netherlands; ^2^ MTA-SZTE Biomimetic Systems Research Group Eötvös Loránd Research Network (ELKH) Dóm tér 8 6720 Szeged Hungary; ^3^ Institute of Physics University of Pécs Ifjúság útja 6 7624 Pécs Hungary; ^4^ Leiden Institute of Chemistry, Gorlaeus Laboratories Leiden University Einsteinweg 55 2333 CC Leiden The Netherlands

**Keywords:** cooperativity, energy decomposition analysis, halogen bonding, MO theory, non-covalent interactions

## Abstract

Cooperative properties of halogen bonds were investigated with computational experiments based on dispersion‐corrected relativistic density functional theory. The bonding mechanism in linear chains of cyanogen halide (X−CN), halocyanoacetylene (X−CC−CN), and 4‐halobenzonitrile (X−C_6_H_4_−CN) were examined for X = H, Cl, Br, and I. Our energy decomposition and Kohn‐Sham molecular‐orbital analyses revealed the bonding mechanism of the studied systems. Cyanogen halide and halocyanoacetylene chains possess an extra stabilizing effect with increasing chain size, whereas the 4‐halobenzonitrile chains do not. This cooperativity can be traced back to charge separation within the σ‐electronic system by charge‐transfer between the lone‐pair orbital of the nitrogen (σ_HOMO_) on one unit and the acceptor orbital of the C−X (σ*_LUMO_) on the adjacent unit. As such, the HOMO‐LUMO gap in the σ‐system decreases, and the cooperativity increases with chain length revealing the similarity in the bonding mechanisms of hydrogen and halogen bonds.

## Graphical abstract

**Longer is stronger**! Halogen bonds in linear chains become increasingly stronger when the chain increases in size. The origin of this cooperativity effect can be traced back to the enhanced donor‐acceptor charge transfer interaction between the units in the chain.

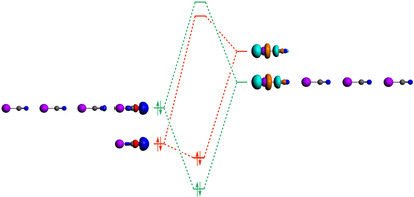

## Introduction

The design and synthesis of supramolecular materials, based on molecular recognition or self‐assembly increasingly attracted the interest of chemists in the past decades.[^1^, ^2^, ^3^] These supramolecular structures are connected by different weak intermolecular interactions, such as hydrogen bonds or π‐π stacking. Recently, halogen bonds have shown to be a new tool in the design of novel complexes and, therefore, its use in supramolecular synthesis increased drastically, due to its directionality and the possibility of modifying its strength in a controlled fashion.[^4^, ^5^, ^6^, ^7^, ^8^]

The simplest halogen bond containing supramolecular systems are one‐dimensional chains, which can be a homomeric system, *i. e*., self‐assembly of units bearing both a halogen bond donor and acceptor site. Otherwise, the chain can be built up of ditopic halogen bond donor and acceptor units, known as, heteromeric systems.[^9^, ^10^, ^11^, ^12^, ^13^] In these systems, the activated chlorine, bromine, and iodine atoms act as strong halogen bond donors and the acceptor at the neighboring unit provides the link for the formation of a one‐dimensional chain. Homomeric chains are nearly linear, consistent with the high directionality of the halogen bond, whereas the heteromeric systems can either exist as a linear or nonlinear chain.

The origin of the bonding mechanism in halogen bonds has been analyzed with different theoretical models and their similarity to hydrogen bonds has been repeatedly demonstrated.[^14^, ^15^, ^16^, ^17^, ^18^, ^19^, ^20^, ^21^, ^22^, ^23^] In this work, we use the energy decomposition analysis (EDA) with the accompanying Kohn‐Sham molecular orbitals (KS‐MO) and Voronoi Deformation Density (VDD) charges to analyze the halogen‐bonded linear chains based on relativistic dispersion‐corrected density functional theory computations.[^24^, ^25^, ^26^, ^27^, ^28^, ^29^, ^30^] Previously, Wolters and Bickelhaupt showed that a covalent interaction exists in halogen‐bonded dimers, similar to dimerization based on hydrogen bonds.^[19]^ Later, Head‐Gordon and coworkers arrived, with the use of an energy decomposition analyses based on absolutely localized molecular orbitals (ALMO‐EDA), at similar conclusions, showing that the charge transfer is indeed responsible for the trend in halogen bond strength.^[23]^ The resemblance with hydrogen bonds has been further explored in tetramers of haloamines and *N*‐halo‐guanines.[^31^, ^32^, ^33^, ^34^] When a quartet was built up from its units, a cooperative effect was found originating from the charge separation that goes with the donor‐acceptor orbital interaction in the σ‐electron system. Additionally, this cooperative effect was found in a variety of other halogen‐bonded systems, containing more than two units.[^35^, ^36^, ^37^, ^38^]

**Figure 1:**
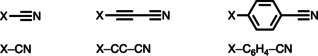
The three building blocks cyanogen halide (X−CN), halocyanoacetylene (X−CC−CN), and 4‐halobenzonitrile (X−C_6_H_4_−CN), with X = H, Cl, Br, or I.

**Scheme 1:**
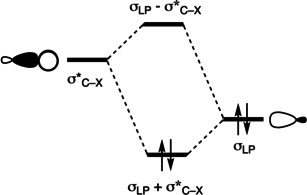
Donor‐acceptor orbital interaction between the C−X antibonding orbital of one fragment, where X = H, Cl, Br, or I, and the lone pair orbital of the nitrogen atom of the other fragment.

The present theoretical work will focus on the bonding mechanism of halogen bonds in linear chains of three different homomeric systems and the presence, or absence, of cooperativity therein. The examined building blocks are cyanogen halide (X−CN), halocyanoacetylene (X−CC−CN), and 4‐halobenzonitrile (X−C_6_H_4_−CN), which we gradually elongate from a dimer to a dodecamer (Figure 1). These monomers vary in their linker between the halogen and the nitrile: cyanogen halide has no linker, *i. e*., the halogen is directly bonded to the nitrile group, whereas in 4‐halocyanoacetylene, the nitrile group is linked to the halogen by acetylene linker, and in 4‐halobenzonitrile the halogen and nitrile group are linked by an aromatic spacer, viz. benzene. We excluded the systems with X = F because previous studies have shown that the fluorine‐containing halogen bonds are not stable.[^31^, ^32^, ^33^, ^34^] We investigate if the lone pair of the nitrogen atom (σ_HOMO_) of one fragment can form a weak donor‐acceptor interaction with the antibonding σ*‐orbital of the C−X on the other fragment (σ*_LUMO_), see Scheme 1. The results of the halogen‐bonded systems are compared to their hydrogen‐bonded analog to proof the intrinsic resemblance between hydrogen bonds and halogen bonds in linear chains.

## Results and Discussion

**Figure 2:**
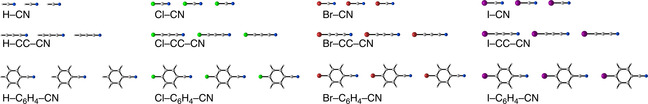
Geometries at ZORA‐BLYP−D3(BJ)/TZ2P for X−CN trimer, X−CC−CN trimer, and X−C_6_H_4_−CN trimer, with X = H, Cl, Br or I. Visualized using CYLview.^[39]^

The infinite chain geometries of cyanogen halide (X−CN), halocyanoacetylene (X−CC−CN), and 4‐halobenzonitrile (X−C_6_H_4_−CN) were calculated with the BAND module using periodic boundary conditions (Figure 2). Taking the specific symmetry of each system into account allows us to mimic the presence of any surface substrate^[8]^ and ensures the perfect separation between the σ‐ and π‐orbital interactions. We, therefore, optimized and analyzed the X−CN and X−CC−CN chains under *C*_∞v_ and X−C_6_H_4_−CN under *C*_s_ symmetry. The halogen bond distances (defined as the distance between X and N) and the halogen bond interaction energies are presented in Table 1. For the X−CN and X−CC−CN infinite chains, the longest halogen bond is found for the X = Cl, 2.87 Å and 2.91 Å respectively, and the shortest in the chains with bromine (2.75 Å and 2.81 Å). On the other hand, for X−C_6_H_4_−CN the longest halogen bond is found for the iodine and the shortest in the chlorine chain, namely, 3.16 Å and 3.11 Å.

**Table 1:** The halogen bond distances **d** [in Å], total interaction energies Δ*E*_int_ [in kcal mol^−1^] for the dimer and dodecamer and the average bond strength in the dodecamer.^[a,b]^

System	X	**d**[X−N]	Δ*E*_int_^dimer^	Δ*E*_int_^dodecamer^	Δ*E*_int_^dodecamer^ /11
X−CN	H	2.00	−4.5	−72.1	−6.6
	Cl	2.87	−3.1	−44.4	−4.0
	Br	2.75	−4.8	−71.5	−6.5
	I	2.81	−6.6	−102.2	−9.3
X−CC−CN	H	2.19	−4.6	−60.6	−5.5
	Cl	2.91	−3.6	−47.0	−4.3
	Br	2.81	−5.3	−71.8	−6.5
	I	2.88	−7.0	−95.2	−8.7
X−C_6_H_4_−CN	H	3.56	−1.5	−17.9	−1.6
	Cl	3.11	−1.2	−15.2	−1.4
	Br	3.12	−2.4	−29.3	−2.7
	I	3.16	−3.6	−44.3	−4.0

[a] Energies computed at ZORA‐BLYP‐D3(BJ)/TZ2P. For X−CN and X−CC−CN chains *C*_∞v_ symmetry has been employed and for X−C_6_H_4_−CN *C*_S_ symmetry. [b] Total interaction energies of all the systems can be found in Table S2.

To analyze the trend in halogen bond lengths along the systems and halogens, we applied the energy decomposition analysis (EDA)[^24^, ^25^, ^26^, ^27^] on the halogen‐bonded dimer and projected the corresponding energy values onto the halogen‐nitrogen distance (Figure S1). This analysis method displayed that the Pauli repulsion is responsible for the longer X−C_6_H_4_−CN halogen bond compared to its X−CN and X−CC−CN counterparts. The Pauli repulsion of the former rises much sharper compared to the latter two when the halogen bond distance is reduced from 3.30 Å to 2.70 Å. Furthermore, the halogen bond of the X−C_6_H_4_−CN system becomes longer when going from Cl to Br to I, due to its increase in atomic radii and, consequently, larger Pauli repulsion. Contrarily, the halogen bond lengths of the X−CN and X−CC−CN systems follow a different trend, because the increasing steric (Pauli) repulsion, due to a larger atomic radius, can partially be compensated by the attractive components of the corresponding stronger halogen bonds.

In Table 1 we presented the total interaction energies of the systems in the dimer and dodecamer chains for the three different linkers. Comparing the total interaction energies reveals that the X−CC−CN systems have the strongest bond, whereas the weakest interactions occur in the case of X−C_6_H_4_−CN. Furthermore, the systems containing the heaviest halogen have the strongest interaction, which is in line with results from previous studies.[^19^, ^32^] The average halogen and hydrogen bond in the dodecamer, *i. e*., Δ*E*_int_^dodecamer^ divided by its 11 bonds, is stronger than the halogen and hydrogen bond in the dimer. In the case of X−CN and X−CC−CN, the average halogen and hydrogen bond is significantly strengthened compared to their analogs in the dimer, which indicates a stabilizing effect on the bonding between the monomers. The average bond strength in the X−C_6_H_4_−CN dodecamer systems is, on the contrary, barely stronger than the dimer interaction, implying the absence of cooperativity in the systems containing a phenyl‐linker. Additionally, the interaction of halogen‐bonded chains containing iodine become more stabilizing than the systems containing chlorine or bromine, showing a more significant cooperative effect in iodine‐based halogen bonds than their chlorine or bromine counterparts.

**Figure 3:**
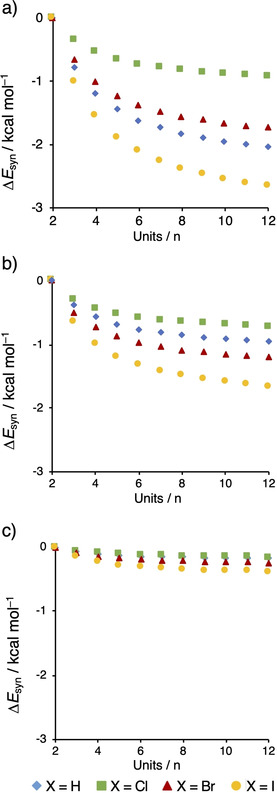
Average synergy of a) (X−CN)_n_, b) (X−CC−CN)_n_, and (X−C_6_H_4_−CN)_n_, where every unit is a separate fragment, the number of units n is displayed on the x‐axis, and the average synergy on the y‐axis. Computed at ZORA‐BLYP‐D3(BJ)/TZ2P. See Figure S2 for the total synergy of the chains, defined as Δ*E*_syn_=Δ*E*_int_^chain^−n ⋅ Δ*E*_int_^dimer^.

To visualize the trend in cooperativity, we calculated the average synergy of the different chains according to Equation (5), while elongating them from two to twelve units (Figure 3). The average synergy is a tool to quantify how much the average halogen and hydrogen bond strengthens when the chain increases in size. For the X−CN and X−CC−CN chains, the average synergy increases when the chain lengthens from a dimer to a dodecamer, which proves cooperativity in these systems. The slope of the Δ*E*_syn_ curves, however, flattens upon adding units to the chain and the synergy closely approaches its asymptotic value around twelve units. Furthermore, the cooperativity of the iodine‐containing halogen‐bonded systems is stronger than that of the hydrogen‐bonded analogs. For all X−C_6_H_4_−CN systems, on the contrary, the average synergy values are almost negligible and, therefore, irrelevant for the structural properties of the system.

To detect the origin of the cooperativity, we applied the energy decomposition analysis (EDA)[^24^, ^25^, ^26^, ^27^] on the chains and calculated the average synergy corresponding to these terms. The average synergy of each energy component, for example, Δ*E*_syn,oi_, is defined in analogy with equation (5): Δ*E*_syn,oi_=(Δ*E*_oi_^chain^/(n−1))−Δ*E*_oi_^dimer^. These results are collected in the Supporting Information Table S3–S5. The two main contributors to the average synergy are the electrostatic interaction (Δ*V*_syn,elstat_) and the orbital interaction (Δ*E*_syn,oi_). Both originate from the charge transfer between occupied and unoccupied orbitals (*vide infra*). The mechanism of enhancement in the halogen bonds is similar to the mechanism in hydrogen bonds as we showed previously for guanine quartets and chains.[^31^, ^32^, ^33^] Although the guanine quartets experience a larger cooperativity effect, chains of guanines also show an enhancement of the interaction energy via the charge separation induced by the donor‐acceptor interactions in the hydrogen bonds.

We can confirm these findings by examining the decomposed energy terms of chains formed by a monomer, dimer, and trimer plus one additional monomer, yielding a dimer, trimer, and tetramer, respectively, where the new monomer is added at the hydrogen or halogen bond acceptor side of the chain (Table 2). The results show that in the cooperative systems (X−CN and X−CC−CN) the Δ*V*_elstat_ and Δ*E*_oi_ increase when the chain lengthens, while in the non‐cooperative systems (X−C_6_H_4_−CN) these energy terms hardly increase. By using symmetry, we found that the largest contribution to the orbital interaction comes from the σ‐electron system and only a minor part from the π‐electron system, which is similar to the hydrogen‐bonded systems.[^40^, ^41^] Moreover, the orbital interactions in the σ‐electron system becomes, when going from chlorine to iodine halogen bonds, more important due to a lower acceptor orbital on the halogen‐donating fragment (*vide infra*). Note that similar results are found when the chain elongates by adding a new monomer to the hydrogen or halogen bond donor side of the chain (Tables S9–S11).

**Table 2:** Energy decomposition [in kcal mol^−1^] of ▵*E*_int_ between the chain (n−1) and an additional monomer for X−CN, X−CC−CN and X−C_6_H_4_−CN with X = H, Cl, Br and I, where the additional monomer is added at the hydrogen or halogen bond acceptor side of the chain.^[a,b,c]^

System	n	Δ*E*_Pauli_	Δ*V*_elstat_	Δ*E*_oi_	Δ*E*_σ_	Δ*E*_π_	Δ*E*_disp_	Δ*E*_int_
H−CN	1+1	8.9	−8.0	−4.3	−3.9	−0.5	−1.1	−4.3
	2+1	9.1	−9.5	−4.7	−4.2	−0.6	−1.1	−6.1
	3+1	9.2	−9.9	−4.8	−4.2	−0.6	−1.1	−6.6
Cl−CN	1+1	5.8	−5.4	−2.2	−1.8	−0.4	−1.3	−3.1
	2+1	5.9	−6.0	−2.4	−2.0	−0.5	−1.3	−3.8
	3+1	5.9	−6.2	−2.5	−2.0	−0.5	−1.3	−4.0
Br−CN	1+1	11.8	−9.5	−5.3	−4.4	−0.9	−1.8	−4.8
	2+1	12.2	−10.7	−5.8	−4.7	−1.0	−1.8	−6.1
	3+1	12.3	−11.1	−5.9	−4.9	−1.0	−1.8	−6.5
I−CN	1+1	15.9	−12.8	−7.5	−6.0	−1.5	−2.3	−6.6
	2+1	16.5	−14.6	−8.2	−6.7	−1.6	−2.3	−8.5
	3+1	16.6	−15.2	−8.5	−6.9	−1.6	−2.3	−9.3
H−CC−CN	1+1	4.7	−5.6	−2.7	−2.2	−0.5	−1.0	−4.6
	2+1	4.8	−6.3	−2.9	−2.3	−0.5	−1.0	−5.3
	3+1	4.8	−6.5	−2.9	−2.3	−0.6	−1.0	−5.5
Cl−CC−CN	1+1	5.1	−5.3	−2.1	−1.6	−0.5	−1.2	−3.6
	2+1	5.2	−5.8	−2.2	−1.7	−0.6	−1.2	−4.1
	3+1	5.2	−5.9	−2.3	−1.7	−0.6	−1.2	−4.3
Br−CC−CN	1+1	9.5	−8.7	−4.5	−3.5	−1.0	−1.7	−5.4
	2+1	9.7	−9.5	−4.8	−3.7	−1.1	−1.7	−6.3
	3+1	9.7	−9.7	−4.8	−3.7	−1.1	−1.7	−6.5
I−CC−CN	1+1	13.2	−11.8	−6.3	−4.8	−1.6	−2.1	−7.0
	2+1	13.4	−12.9	−6.8	−5.1	−1.7	−2.1	−8.3
	3+1	13.5	−13.2	−6.9	−5.2	−1.7	−2.1	−8.7
H−C_6_H_4_−CN	1+1	0.2	−1.2	−0.2	−0.1	−0.1	−0.3	−1.5
	2+1	0.2	−1.3	−0.2	−0.1	−0.1	−0.3	−1.6
	3+1	0.2	−1.3	−0.2	−0.1	−0.1	−0.3	−1.6
Cl−C_6_H_4_−CN	1+1	3.0	−1.9	−1.1	−0.9	−0.2	−1.1	−1.2
	2+1	3.0	−2.0	−1.2	−1.0	−0.3	−1.1	−1.4
	3+1	3.0	−2.0	−1.2	−1.0	−0.3	−1.1	−1.4
Br−C_6_H_4_−CN	1+1	4.2	−3.3	−1.9	−1.7	−0.2	−1.4	−2.4
	2+1	4.2	−3.4	−2.0	−1.8	−0.2	−1.4	−2.6
	3+1	4.2	−3.4	−2.0	−1.8	−0.2	−1.4	−2.7
I−C_6_H_4_−CN	1+1	6.3	−5.3	−2.8	−2.5	−0.3	−1.9	−3.6
	2+1	6.4	−5.5	−2.9	−2.6	−0.3	−1.9	−4.0
	3+1	6.4	−5.6	−3.0	−2.6	−0.3	−1.9	−4.0

[a] Energies computed at ZORA‐BLYP‐D3(BJ)/TZ2P. [b] The chain is fragmented into two fragments: (i) the chain with length n−1 and (ii) one additional unit. [c] Complete table with the data up to the dodecamer can be found in the Supporting Information Table S6–S8.

Analysis of the charge transfer confirms this picture and provides a straightforward explanation for the cooperativity in halogen‐bonded chains: the donor‐acceptor interaction, between the unoccupied σ*_C−X_ orbital of the monomer and nitrogen lone‐pair σ_LP_ orbital of the chain, associated with the Δ*E*_σ_ term induces a charge separation, which, in turn, enhances both the orbital interaction and the electrostatic interaction. The number of electrons that are donated into the σ*_LUMO_ of the halogen bond donor of the X−CN series is for H, Cl, Br, and I, 0.03, 0.03, 0.05, and 0.07 electrons, respectively. In the X−CC−CN series the donation is 0.02 electrons for H, 0.02 electrons for Cl, 0.04 electrons for Br, and 0.06 electrons for I. In the X−C_6_H_4_−CN series fewer electrons are donated to the σ*_LUMO_ of the halogen bond donor: 0.00, 0.01, 0.02, and 0.03 electrons, for respectively X = H, Cl, Br, and I. The number of electrons that is donated into the σ*_LUMO_ of the halogen bond donor remains constant throughout lengthening of the chain.

**Figure 4:**
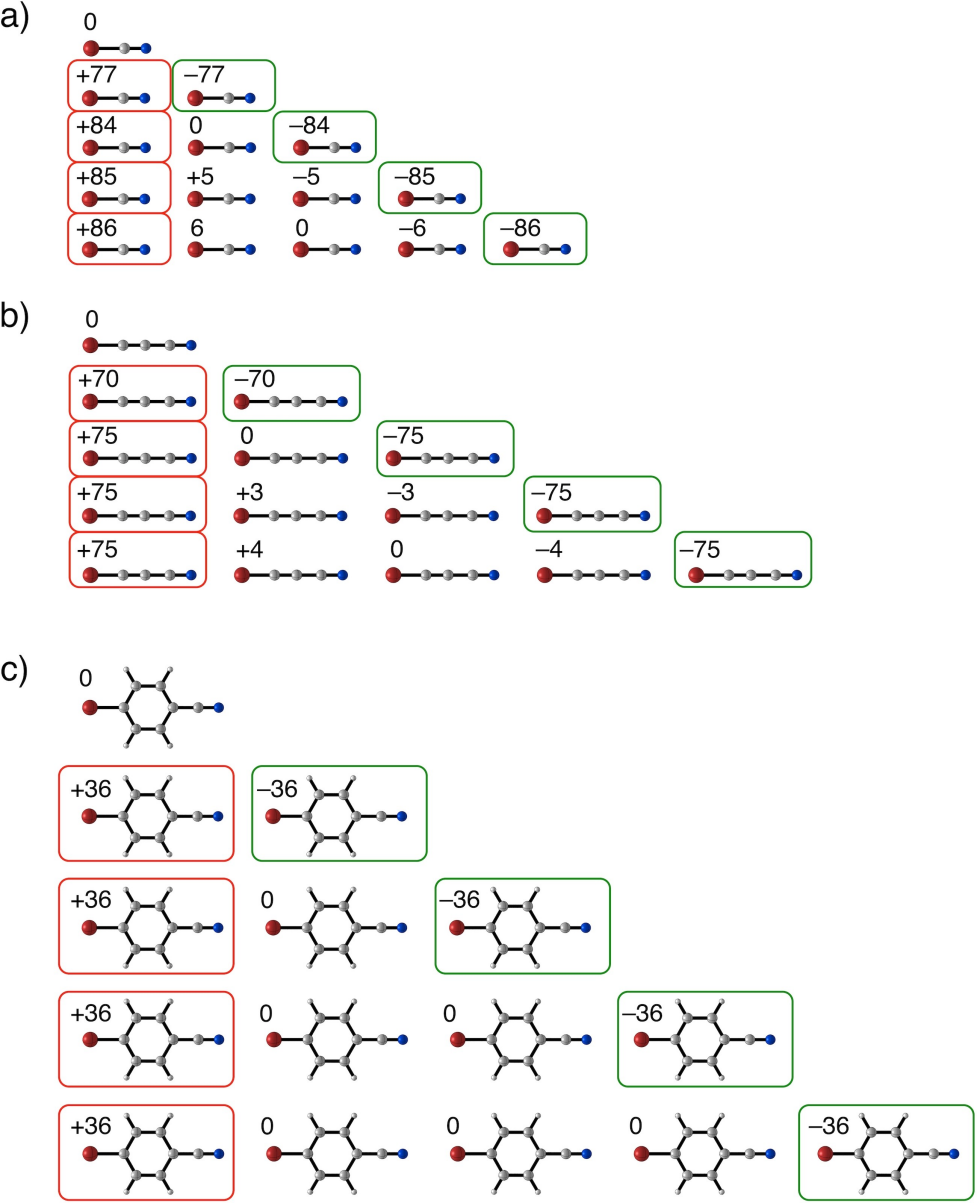
Examples of VDD atomic charges [in milli‐electrons] for the units of cooperative systems a) Br−CN, b) Br−CC−CN, and non‐cooperative system c) Br−C_6_H_4_−CN computed at ZORA‐BLYP‐D3(BJ)/TZ2P.

This charge transfer in the σ‐system of the chains can be quantified with the VDD charge analysis. For all the halogen‐ and hydrogen‐bonded systems, the unit at the acceptor side of the chain has a positive VDD charge because the charge will flow from this unit, upon bonding, towards the donor side of the chain (Figure 4). In the cooperative systems, this σ‐donor‐acceptor interaction leads to an increasing charge separation, which can be seen for Br−CN and Br−CC−CN. For n = 2, the charge of the first unit amounts +77 and +70 milli‐electrons respectively, whereas, for n = 5, the respective charges of the first unit are +86 and +75 milli‐electrons. Nevertheless, for non‐cooperative systems, the charge separation remains constant when the chain lengthens, namely, +36 milli‐electrons.

**Figure 5:**
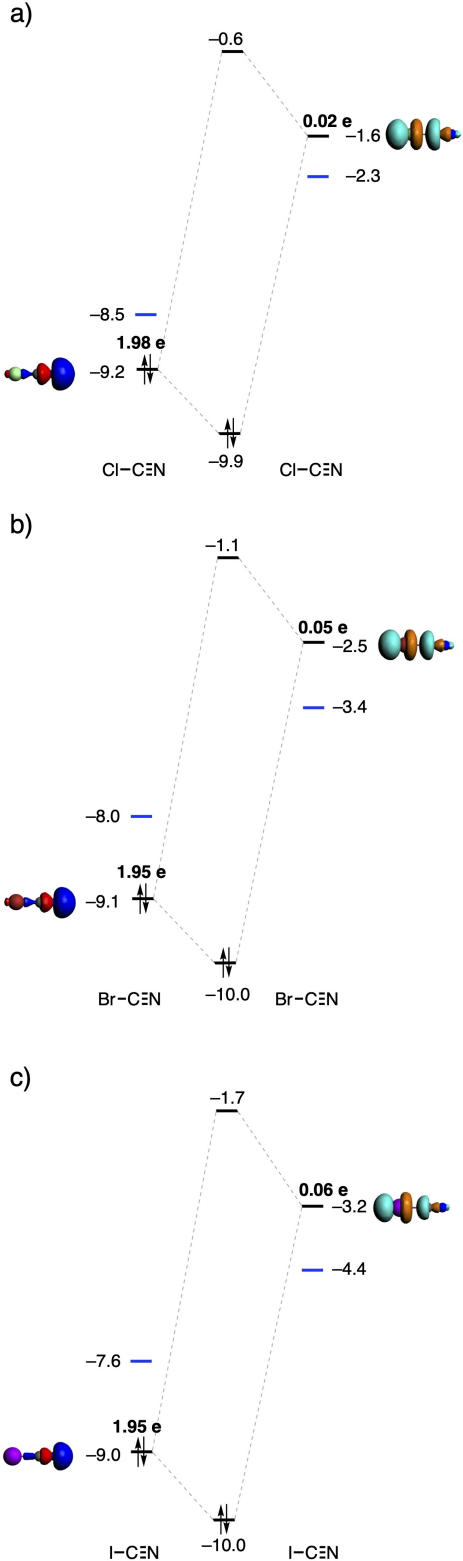
Molecular orbital diagram of the formation of a) Cl−CN dimer; b) Br−CN dimer; and c) I−CN dimer, where the orbital energies are displayed in eV. The blue energy levels show the HOMO and LUMO of two interacting dodecamers.

The Kohn‐Sham molecular orbital analysis confirmed that the main donor‐acceptor interaction of all the halogen‐bonded systems is between the σ_HOMO_ of the nitrogen and the σ*_LUMO_ of the halogen (Figure 5).[^29^, ^30^] The black MO diagram shows the donor‐acceptor interaction between the σ_HOMO_ and σ*_LUMO_ of two monomers, and the blue energy levels are the σ_HOMO_ and σ*_LUMO_ of two interacting dodecamers. The stability of the σ*_LUMO_ orbital of the C−X bond lowers significantly when we vary the system from X−CN or X−CC−CN to X−C_6_H_4_−CN, for instance, for X = Br, the σ*_LUMO_ orbital goes from −2.5 eV to −2.3 eV to −1.6 eV, for Br−CN to Br−CC−CN to Br−C_6_H_4_−CN, respectively (Table 3), which is in line with our previous work.^[42]^ In that paper, we showed that the σ*_LUMO_ of a C−X bond becomes more stable when we go from an sp^2^‐hybridized C−X bond to an sp‐hybridized C−X bond, because the SOMO of the sp^2^‐hybridized carbon radical, which participates in the electron‐pair bond formation with X yielding the C−X bond, is more stable than the sp‐hybridized analog. Furthermore, σ*_LUMO_ becomes more stable when the C−X bond contains a larger halogen due to the more diffuse halogen *n*p_σ_ atomic orbital which gives rise to a decrease in the overlap of the antibonding combination of the carbon 2 s and halogen *n*p_σ_ atomic orbital (AO).^[43]^ The HOMO‐LUMO gaps of all interactions clearly become smaller when the chain lengthens. The energy gap of the X−CN systems decreases 1.8 eV, 1.5 eV, 2.1 eV and 2.5 eV for respectively X = H, Cl, Br, and I. In the X−CC−CN systems the energy gap decreases 1.1 eV for X = H, 1.0 eV for X = Cl, 1.3 eV for X = Br, and 1.5 eV for X = I. At last, in the X−C_6_H_4_−CN systems the energy gap decreases less than the former two systems, namely, 0.1 eV, 0.3 eV, 0.4 eV, and 0.6 eV, for X = H, Cl, Br and I, respectively. The larger decrease of HOMO‐LUMO energy gap in the cooperative systems can be explained by the increasing charge separation because the net negative charge on the latter unit leads to a destabilization of the nitrogen lone‐pair orbital; and the net positive charge on the former unit stabilizes the energy level of the acceptor orbital of the carbon‐halogen/hydrogen bond. Furthermore, the energy gap between the σ_HOMO_ of the nitrogen and the σ*_LUMO_ of the X = I is in all cases smaller than the X = H, Cl or Br (I<Br<Cl<H), which explains why the iodine systems have more cooperativity than the hydrogen, bromine and chlorine systems.

**Table 3:** The energy levels of the HOMO and LUMO together with the corresponding HOMO‐LUMO energy gap [in eV] of two interacting monomers and dodecamers for X−CN, X−CC−CN, and X−C_6_H_4_−CN with X = H, Cl, Br and I.^[a]^

Systems	X	HOMO		LUMO		Energy Gap	
		1	12	1	12	1+1	12+12
X−CN	H	−9.1	−8.0	0.0	−0.8	−9.1	−7.3
	Cl	−9.2	−8.5	−1.6	−2.3	−7.6	−6.2
	Br	−9.1	−8.0	−2.5	−3.4	−6.7	−4.6
	I	−9.0	−7.6	−3.2	−4.4	−5.8	−3.3
X−CC−CN	H	−9.2	−8.6	−0.1	−0.6	−9.1	−7.9
	Cl	−9.0	−8.5	−1.3	−1.7	−7.8	−6.7
	Br	−9.0	−8.4	−2.3	−2.9	−6.7	−5.4
	I	−9.0	−8.2	−2.9	−3.6	−6.1	−4.6
X−C_6_H_4_−CN	H	−8.5	−8.4	0.4	−0.4	−8.1	−8.0
	Cl	−8.6	−8.4	−0.9	−1.0	−7.7	−7.4
	Br	−8.6	−8.4	−1.6	−1.8	−7.0	−6.6
	I	−8.6	−8.3	−2.3	−2.6	−6.3	−5.7

[a] Energies computed at ZORA‐BLYP‐D3(BJ)/TZ2P.

Thus, cooperativity becomes more pronounced every time an additional unit is added to the chain because such addition will amplify the charge separation, and thereby the donor‐acceptor interactions. Cooperativity only occurs in the X−CN and X−CC−CN systems, which is showed by their substantial amount of average synergy and the increasing charge separation when the systems lengthen. The cooperative effect stays constant when the chain gets longer than twelve units.

## Conclusion

The theoretical experiment presented in this work, in which we compared the halogen and hydrogen bonds in three different linear systems with different halogen/hydrogen‐nitrile linkers, demonstrates that cooperativity occurs in all X−CN and X−CC−CN systems. Furthermore, all the X−C_6_H_4_−CN systems did not show a cooperative effect, due to an initially large HOMO‐LUMO gap which cannot be overcome by the weak charge transfer. The cooperativity in halogen‐bonded systems was proven by the charge separation, which arises from the donor‐acceptor orbital interaction in the σ‐electron system of the lone‐pair orbital of nitrogen (σ_HOMO_) on one unit and the acceptor orbital of the halogen (σ*_LUMO_) on the other unit. The cooperativity in the X−CN and X−CC−CN systems become stronger when a unit is added to the supramolecular system because such an addition leads to an enlargement in the charge separation, and therefore a smaller HOMO‐LUMO gap. These findings were supported by the calculation of the average synergy, which showed that the X−CN and X−CC−CN systems have a significant amount of synergy, and, consequently, are cooperative systems. Notably, this is achieved by using explicit quantities, *i. e*., the interaction energy and the electron density distribution. The Kohn‐Sham MO theory, supported by the quantitative interaction energy decomposition scheme, is used to attain the physical interpretation of the results.

## Computational Method

All calculations were carried out with the Amsterdam Density Functional (ADF) program applying the ADF and BAND modules.[^44^, ^45^, ^46^] We used dispersion‐corrected relativistic density functional theory at the ZORA‐BLYP‐D3(BJ)/TZ2P level of theory for geometry optimization and energies.[^47^, ^48^, ^49^, ^50^, ^51^, ^52^, ^53^] We benchmarked this level of theory using the S22 data set^[54]^ and found that, in line with Head‐Gordon *et al*.,^[55]^ that ZORA‐BLYP−D3(BJ)/TZ2P accurately describes weak interactions (Tables S13–15). Infinite chain geometries were optimized applying the BAND module and finite ground‐state (closed‐shell) fragments were cut out with a length of two till twelve units. (Full computational details are available in the Supporting Information.)

The bond energy Δ*E*_bond_ of the chain with n units is defined as [Eq. 1]:

(1) $$\Delta E{}_{\mathrm{bond}}=E{}_{\mathrm{chain}}-n\;\cdot \;E{}_{\mathrm{unit}}$$

where *E*_chain_ is the energy of the chain, *E*_unit_ is the energy of a single monomer unit in its equilibrium geometry, and n is the number of units in the chain. The overall bond energy Δ*E*_bond_ is made up of two major components [Eq. 2]:

(2) $$\Delta E{}_{\mathrm{bond}}=\Delta E{}_{\mathrm{prep}}+\Delta E{}_{\mathrm{int}}$$

In this formula, the preparation energy Δ*E*_prep_ is the amount of energy that is required to deform the constituent units from their equilibrium structure to the geometry they acquire in the chain and the interaction energy Δ*E*_int_ accounts for all the chemical interactions that occur between the units in a chain.

The interaction energy in the systems is examined in the framework of the Kohn‐Sham molecular orbital model using a quantitative energy decomposition analysis (EDA), which decomposes the interaction energy into electrostatic interaction, Pauli repulsion, orbital interaction, to which a term Δ*E*_disp_ is added to account for the dispersion interaction [Eq. 3]:[^24^, ^25^, ^26^, ^27^]

(3) $$\Delta E{}_{\mathrm{int}}=\Delta V{}_{\mathrm{elstat}}+\Delta E{}_{\mathrm{Pauli}}+\Delta E{}_{\mathrm{oi}}+\Delta E{}_{\mathrm{disp}}$$

The electrostatic energy Δ*V*_elstat_ is the classical Coulomb interaction between the unperturbed charge distributions of the prepared, *i. e*., deformed, units and is usually attractive. The Pauli repulsion Δ*E*_Pauli_ comprises the destabilizing interaction between occupied orbitals and is responsible for any steric repulsion. The orbital interaction energy Δ*E*_oi_ accounts for the polarization (empty‐occupied orbital mixing on one fragment due to the presence of another fragment) and charge transfer (donor‐acceptor interactions between occupied orbitals on one fragment and unoccupied orbitals on the other, including the HOMO‐LUMO interactions). The dispersion energy Δ*E*_disp_ accounts for the dispersion corrections as introduced by Grimme *et al*.^[47]^

The orbital interaction energy can be further decomposed into the contributions from each irreducible representation of the interacting system (Equation (4)).^[56]^ In our model systems with a mirror plane parallel to the halogen bonds, we can distinguish σ and π orbital interactions: [Eq. 4]

(4) $$\Delta E{}_{\mathrm{oi}}=\Delta E{}_{\sigma }+\Delta E{}_{\pi }$$

The average synergy, a measure to quantify the amount of cooperativity in the linear systems, is determined by comparing the average total interaction energy of the linear chain containing n units with the total interaction energy of a dimer [Eq. 5].

(5) $$\Delta E{}_{\mathrm{syn}}=\left(\Delta E{}_{\mathrm{int}}{}^{\mathrm{chain}}/\left(n-1\right)\right)-\Delta E{}_{\mathrm{int}}{}^{\mathrm{dimer}}$$

Here, Δ*E*_int_^chain^ is the total interaction energy of the linear chain with n units and Δ*E*_int_^dimer^ is the total interaction energy of the dimer. A negative value of Δ*E*_syn_ corresponds to a positive cooperative effect in the chain, which reinforces the average total interaction energy.

The Voronoi Deformation Density (VDD) charge *Q*_A_ is calculated as the (numerical) integral of the deformation density in the volume of the Voronoi cell of atom A (Equation (6)).^[28]^ The Voronoi cell of atom A is determined as the compartment of space between the bond midplanes on and perpendicular to all bond axes between nucleus A and the neighboring nuclei.

(6) $$Q_{A}^{\mathrm{VDD}}=-\underset{\mathrm{Voronoi}\;\mathrm{cell}\;\mathrm{of}\;A}{\int^{\;}}[\rho \left(r\right)-\rho_{\mathrm{promolecule}}\left(r)\right]dr\;$$

In this formula, *ρ*(**r**) is the electron density of the supramolecular system and Σ_B_ *ρ*_B_(**r**) is the sum of the atomic densities *ρ*_B_ of a neutral atom without any chemical interaction. The VDD charge *Q*_A_ does not measure the amount of charge on a particular atom A, but monitors the charge flow out (*Q*_A_>0) or into (*Q*_A_<0) the Voronoi cell of atom A upon interaction.

## Conflict of interest

The authors declare no conflict of interest.

## Supporting information

As a service to our authors and readers, this journal provides supporting information supplied by the authors. Such materials are peer reviewed and may be re‐organized for online delivery, but are not copy‐edited or typeset. Technical support issues arising from supporting information (other than missing files) should be addressed to the authors.

Supplementary
