# Supplementary material for: Cooperative Self‐Assembly in Linear Chains Based on Halogen Bonds
Source: Chempluschem. 2021 May 6;86(6):812–9. doi: 10.1002/cplu.202100093 (PMC8252609; doi:10.1002/cplu.202100093)
Supplement: Supplementary file 1 — Supplementary [file CPLU-86-812-s001.pdf]

ChemPlusChem

Supporting Information

Cooperative Self-Assembly in Linear Chains Based on Halogen Bonds

Pascal Vermeeren, Lando P. Wolters, Gábor Paragi,* and Célia Fonseca Guerra*

Content

Appendix S1. Full computational details.

Figure S1. Decomposed energy terms [in kcal mol^{-1}] projected onto the halogen-bond distance r [in Å] for X–CN and X–C_6H_4–CN, where X = Cl, Br, and I.

Table S1. Halogen bond lengths [in Å] of Cl–CN, Br–CN, and I–CN computed using BAND and ADF.

Figure S2. Total synergy, defined as $\Delta E_{syn} = \Delta E_{int}^{chain} - n \bullet \Delta E_{int}^{dimer}$, of a) $(X\text{–}CN)_n$, b) $(X\text{–}CC\text{–}CN)_n$, and c) $(X\text{–}C_6H_4\text{–}CN)_n$, where the number of units n is displayed on the x-axis and the total synergy on the y-axis. Computed at ZORA-BLYP-D3(BJ)/TZ2P.

Table S2. Interaction energy [in kcal mol^{-1}] of $(X\text{–}CN)_n$, $(X\text{–}CC\text{–}CN)_n$, and $(X\text{–}C_6H_4\text{–}CN)_n$ with X = H, Cl, Br, and I computed from n units.

Table S3. Energy decomposition analysis of $\Delta\bar{E}_{syn}$ [in kcal mol^{-1}] of $(X\text{–}CN)_n$ with X = H, Cl, Br, and I.

Table S4. Energy decomposition analysis of $\Delta\bar{E}_{syn}$ [in kcal mol^{-1}] of $(X\text{–}CC\text{–}CN)_n$ with X = H, Cl, Br, and I.

Table S5. Energy decomposition analysis of $\Delta\bar{E}_{syn}$ [in kcal mol^{-1}] of $(X\text{–}C_6H_4\text{–}CN)_n$ with X = H, Cl, Br, and I.

Table S6. Energy decomposition [in kcal mol^{-1}] of the ΔE_{int} between the chain of n units and an additional monomer for $(X\text{–}CN)_{n+1}$ with X = H, Cl, Br, and I, where the additional monomer is added at the hydrogen or halogen bond acceptor side of the chain.

Table S7. Energy decomposition [in kcal mol^{-1}] of the ΔE_{int} between the chain of n units and an additional monomer for $(X\text{–}CC\text{–}CN)_{n+1}$ with X = H, Cl, Br, and I, where the additional monomer is added at the hydrogen or halogen bond acceptor side of the chain.

Table S8. Energy decomposition [in kcal mol^{-1}] of the ΔE_{int} between the chain of n units and an additional monomer for $(X\text{–}C_6H_4\text{–}CN)_{n+1}$ with X = H, Cl, Br, and I, where the additional monomer is added at the hydrogen or halogen bond acceptor side of the chain.

Table S9. Energy decomposition [in kcal mol^{-1}] of the ΔE_{int} between the chain of n units and an additional monomer for $(X\text{–}CN)_{n+1}$ with X = H, Cl, Br, and I, where the additional monomer is added at the hydrogen or halogen bond donor side of the chain.

Table S10. Energy decomposition [in kcal mol^{-1}] of the ΔE_{int} between the chain of n units and an additional monomer for $(X\text{–}CC\text{–}CN)_{n+1}$ with X = H, Cl, Br, and I, where the additional monomer is added at the hydrogen or halogen bond donor side of the chain.

Table S11. Energy decomposition [in kcal mol^{-1}] of the ΔE_{int} between the chain of n units and an additional monomer for $(X–C_6H_4–CN)_{n+1}$ with X = H, Cl, Br, and I where the additional monomer is added at the hydrogen or halogen bond donor side of the chain.

Table S12. The VDD charges [in milli-electrons] of the first and last monomers in the $(X–CN)_n$, $(X–CC–CN)_n$ and $(X–C_6H_4–CN)_n$ systems, with X = H, Cl, Br, and I.

Table S13. Binding and interaction energy [in kcal mol^{-1}] and intermolecular distances R [in Å] for several hydrogen-bonded model complexes.

Table S14. Binding and interaction energies [in kcal mol^{-1}] and intermolecular distances R [in Å] for several model van-der-Waals complexes.

Table S15. Binding and interaction energies [in kcal mol^{-1}] and intermolecular distances R [in Å] for other weakly bound complexes.

Table S16. Cartesian coordinates [in Å] of all the infinite X–CN, X–CC–CN, and X–C_6H_4–CN systems computed at ZORA-BLYP-D3(BJ)/TZ2P.

Table S17. Cartesian coordinates [in Å], applied point group symmetry and total ADF bonding energies [in kcal mol^{-1}] of all optimized molecular fragments included in the extended S22 benchmark set, computed at ZORA-BLYP-D3(BJ)/TZ2P.

Table S18. Cartesian coordinates [in Å], applied point group symmetry and total ADF bonding energies [in kcal mol^{-1}] of all optimized molecular complexes included in the extended S22 benchmark set, computed at ZORA-BLYP-D3(BJ)/TZ2P.

Appendix S1. Full computational details.

All the calculations were performed with the Amsterdam Density Functional (ADF) [1,2,3] program applying the ADF and BAND modules.[4] The numerical integration was carried out using the procedure developed by te Velde et al.[5,6] The molecular orbitals were expanded in a large uncontracted set of Slater type orbitals (STOs): TZ2P. The TZ2P basis set [7] has the triple-ξ quality for all atoms and has been enhanced with two sets of polarization functions, that is, 2p and 3d on H; 3d and 4f on carbon, nitrogen, oxygen and chlorine; 4d and 4f on bromine; 5d and 4f on iodine. No core shells of the atoms were treated by the frozen-core approximation. The molecular density was fitted by the Zlm fitting scheme.[8] Equilibrium structures were attained by optimizations using analytical gradient techniques.[9] Geometries and energies were calculated at the BLYP level of generalized gradient approximation (GGA). BLYP described the exchange by Slater's Xα potential,[10] with nonlocal corrections due to Becke[11,12] added self-consistently, and the gradient-corrected functional of Lee, Yang and Parris used for the correlation.[13,14,15] Dispersion corrections are applied using the DFT-D3(BJ) method, developed by Grimme,[16] which contains the damping function proposed by Becke and Johnson.[17] In this approach, the density functional is augmented with an empirical term correcting for long-range dispersion effects, described by a sum of damped interatomic potentials of the form $C_6/(R^6+c)$ added to the usual DFT energy. Scalar relativistic effects were accounted for using the zeroth-order regular approximation (ZORA).[18,19]

[1] G. te Velde, F. M. Bickelhaupt, E. J. Baerends, C. Fonseca Guerra, S. J. A. van Gisbergen, J. G. Snijders, T. Ziegler, *J. Comp. Chem.* **2001**, *22*, 931.

[2] C. Fonseca Guerra, J. G.Snijders, G. te Velde, E. J. Baerends, *Theor. Chem. Acc.* **1998**, *99*, 391.

[3] ADF2014, SCM, Theoretical Chemistry, Vrije Universiteit, Amsterdam (The Netherlands), **2014**.

[4] BAND2014, SCM, Theoretical Chemistry, Vrije Universiteit, Amsterdam (The Netherlands), **2014**.

[5] P. M. Boerrigter, G. te Velde, E. J. Baerends, *Int. J. Quantum Chem.* **1988**, *33*, 87.

[6] G. te Velde, E. J. Baerends, *J. Comput. Phys.* **1992**, *99*, 84.

[7] E. van Lenthe, E. J. Baerends, *J. Comput. Chem.* **2003**, *24*, 1142.

[8] M. Franchini, P. H. T. Philipsen, E. van Lenthe, L. Visscher, *J. Chem. Theory Comput.* **2014**, *10*, 1994.

[9] L. Versluis, T. Ziegler, *J. Chem. Phys.* **1988**, *88*, 322.

[10] J. C. Slater, *Quantum Theory of Molecules and Solids, Vol. 4*, McGraw-Hill, New York, **1974**.

[11] A. D. Becke, *J. Chem. Phys.* **1986**, *84*, 4524.

[12] A. D. Becke, *Phys. Rev. A.* **1988**, *38*, 3098.

[13] C. Lee, W. Yang, R. G. Parr, *Phys. Rev. B.* **1988**, *37*, 785.

[14] B. G.Johnson, P. M. W. Gill, J. A. Pople, *J. Chem. Phys.* **1993**, *98*, 5612.

[15] T. V. Russo, R. L. Martin, P. J. Hay, *J. Chem. Phys.* **1994**, *101*, 7729.

[16] S. Grimme, S. Ehrlich, L. Goerigk, *J. Comput. Chem.* **2011**, *32*, 1456.

[17] E. R. Johnson, A. D. Becke, *J. Chem. Phys.* **2005**, *123*, 024101.

[18] E. van Lenthe, E. J. Baerends, J. G. Snijders, *J. Chem. Phys.* **1994**, *101*, 9783.

[19] E. van Lenthe, R. van Leeuwen, E. J. Baerends, J. G. Snijders, *Int. J. Quantum Chem.* **1996**, *57*, 281.

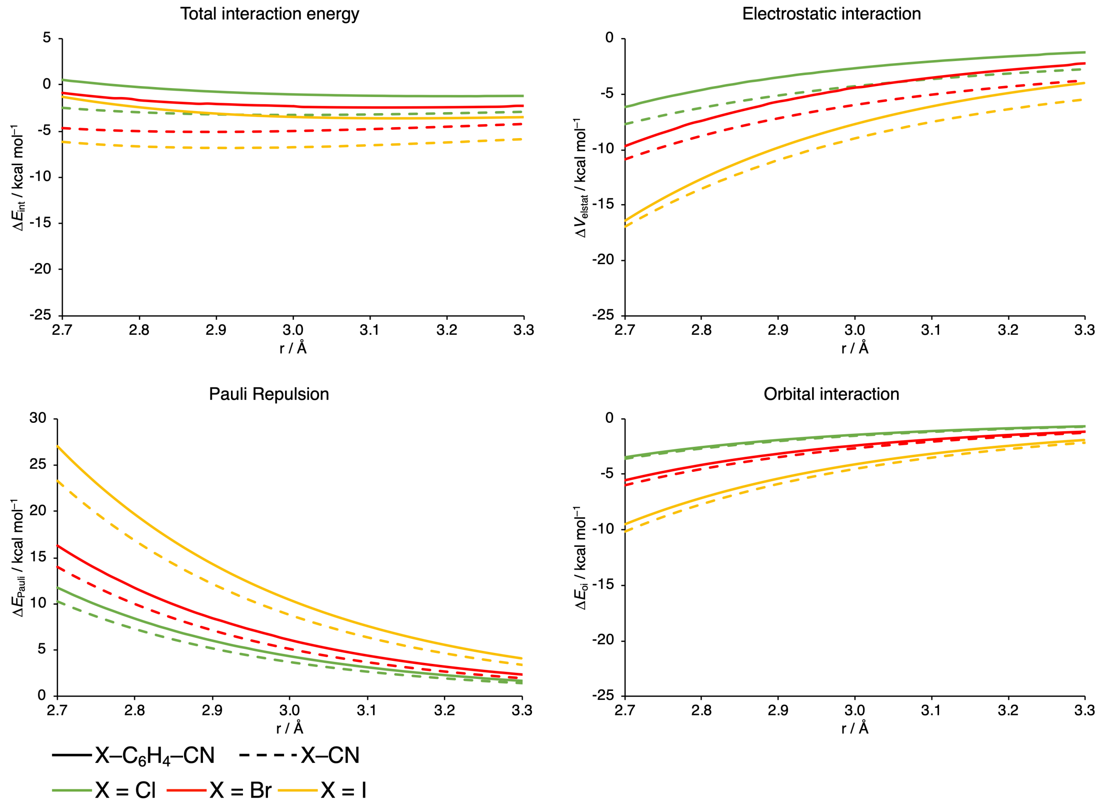

Figure S1. Decomposed energy terms [in kcal mol^{-1}] projected onto the halogen-bond distance r [in Å] for X–CN and X–C_6H_4–CN, where X = Cl, Br, and I. At each point, the dimers are optimized with constrained geometries and fixed halogen bond distances r, computed at ZORA-BLYP-D3(BJ)/TZ2P.

Table S1. Halogen bond lengths [in Å] of Cl–CN, Br–CN, and I–CN computed using BAND and ADF.[a]

System	X	d(X–N) BAND infinite chain optimization	d(X–N) ADF dimer optimization
**X–CN**	Cl	2.87	2.99
	Br	2.75	2.90
	I	2.81	2.93

[a] Energies computed at ZORA-BLYP-D3(BJ)/TZ2P in $C_{\infty v}$ symmetry.

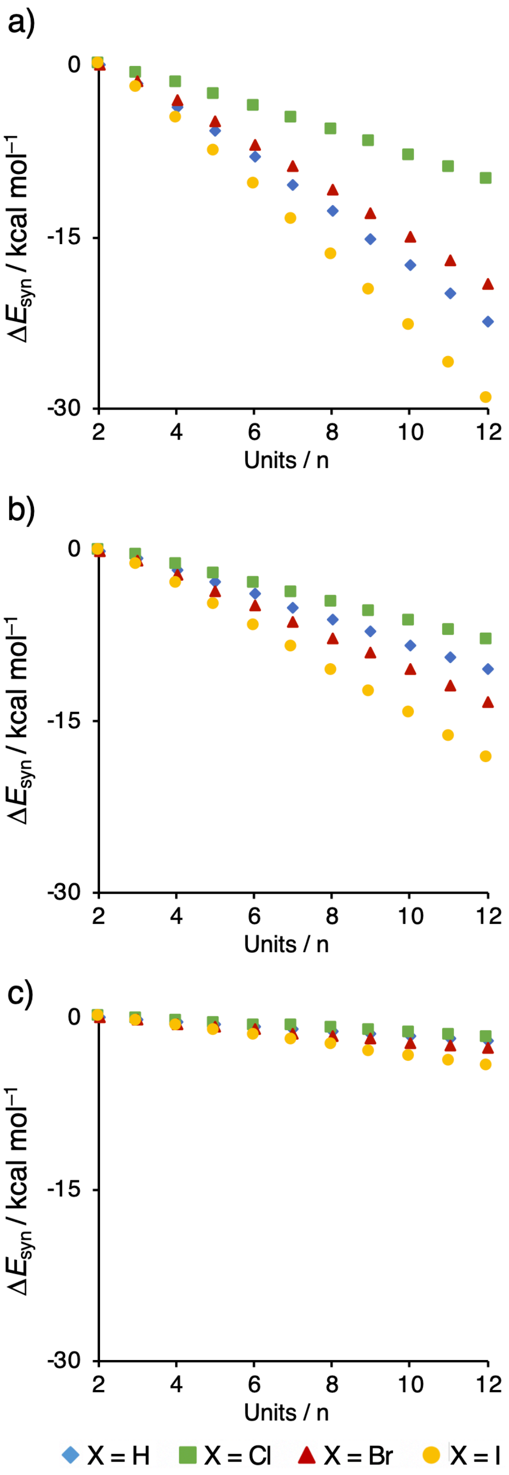

Figure S2. Synergy, defined as $\Delta E_{syn} = \Delta E_{int}^{chain} - n \bullet \Delta E_{int}^{dimer}$, of a) $(X–CN)_n$, b) $(X–CC–CN)_n$, and c) $(X–C_6H_4–CN)_n$, where the number of units n is displayed on the x-axis and the synergy on the y-axis. Computed at ZORA-BLYP-D3(BJ)/TZ2P.

Table S2. Interaction energy [in kcal mol^{-1}] of $(X–CN)_n$, $(X–CC–CN)_n$ and $(X–C_6H_4–CN)_n$ with X = H, Cl, Br,and I computed from n units.[a]

System	n	H	Cl	Br	I
**X–CN**	2	–4.52	–3.12	–4.77	–6.64
	3	–10.6	–6.95	–10.85	–15.28
	4	–17.14	–10.98	–17.34	–24.56
	5	–23.86	–15.10	–23.99	–34.08
	6	–30.67	–19.25	–30.71	–43.71
	7	–37.53	–23.42	–37.47	–53.41
	8	–44.42	–27.60	–44.25	–63.13
	9	–51.32	–31.79	–51.04	–72.88
	10	–58.23	–35.98	–57.85	–82.65
	11	–65.15	–40.18	–64.66	–92.42
	12	–72.10	–44.38	–71.47	–102.20
**X–CC–CN**	2	–4.56	–3.55	–5.32	–6.99
	3	–9.88	–7.68	–11.63	–15.28
	4	–15.40	–11.96	–18.16	–23.94
	5	–20.99	–16.29	–24.79	–32.74
	6	–26.62	–20.66	–31.46	–41.60
	7	–32.27	–25.04	–38.15	–50.50
	8	–37.92	–29.42	–44.86	–59.41
	9	–43.59	–33.82	–51.58	–68.34
	10	–49.26	–38.22	–58.31	–77.28
	11	–54.93	–42.62	–65.04	–86.23
	12	–60.61	–47.03	–71.77	–95.18
**X–C_6H_4–CN**	2	–1.45	–1.13	–1.87	–3.64
	3	–3.05	–2.37	–3.92	–7.60
	4	–4.68	–3.63	–6.00	–11.64
	5	–6.32	–4.90	–8.10	–15.71
	6	–7.97	–6.17	–10.2	–19.79
	7	–9.62	–7.44	–12.31	–23.87
	8	–11.27	–8.72	–14.42	–27.96
	9	–12.92	–9.99	–16.53	–32.05
	10	–14.56	–11.27	–18.64	–36.14
	11	–16.22	–12.55	–20.75	–40.23
	12	–17.86	–13.82	–22.86	–44.33

[a] Energies computed at ZORA-BLYP-D3(BJ)/TZ2P in $C_{\infty v}$ symmetry for X–CN and X–CC–CN and C_S symmetry for X–C_6H_4–CN.

Table S3. Energy decomposition analysis of $\Delta\bar{E}_{syn}$ [in kcal mol^{-1}] of $(X–CN)_n$ with X = H, Cl, Br, and I.[a]

System	n	$\Delta\bar{E}_{syn}$	$\Delta E_{syn,oi}$	$\Delta V_{syn,elstat}$	$\Delta E_{syn,Pauli}$	$\Delta E_{syn,disp}$
**H–CN**	3	–0.78	–0.27	–0.24	0.00	0.00
	4	–1.19	–0.56	–0.36	0.00	0.00
	5	–1.45	–0.74	–0.43	0.00	0.00
	6	–1.61	–0.86	–0.48	0.00	0.00
	7	–1.74	–0.95	–0.52	0.00	0.00
	8	–1.83	–1.01	–0.54	0.00	–0.01
	9	–1.90	–1.06	–0.57	0.01	–0.01
	10	–1.95	–1.10	–0.58	0.01	–0.01
	11	–2.00	–1.13	–0.60	0.01	–0.01
	12	–2.03	–1.16	–0.61	0.01	–0.01
**Cl–CN**	3	–0.36	–0.26	–0.10	0.00	0.00
	4	–0.54	–0.40	–0.15	0.00	–0.01
	5	–0.66	–0.48	–0.18	0.00	–0.01
	6	–0.73	–0.53	–0.20	0.01	–0.01
	7	–0.78	–0.57	–0.21	0.01	–0.01
	8	–0.82	–0.60	–0.22	0.01	–0.01
	9	–0.85	–0.62	–0.23	0.00	–0.01
	10	–0.88	–0.64	–0.24	0.01	–0.01
	11	–0.90	–0.65	–0.24	0.01	–0.01
	12	–0.91	–0.66	–0.25	0.01	–0.01
**Br–CN**	3	–0.66	–0.54	–0.12	–0.01	0.00
	4	–1.01	–0.82	–0.18	–0.01	0.00
	5	–1.23	–1.00	–0.22	–0.02	0.00
	6	–1.37	–1.12	–0.24	–0.02	0.00
	7	–1.48	–1.20	–0.26	–0.02	0.00
	8	–1.55	–1.27	–0.27	–0.02	0.00
	9	–1.61	–1.31	–0.29	–0.02	0.00
	10	–1.66	–1.35	–0.29	–0.02	0.00
	11	–1.70	–1.38	–0.30	–0.02	0.00
	12	–1.73	–1.41	–0.31	–0.02	0.00
**I–CN**	3	–1.00	–0.82	–0.15	–0.03	0.00
	4	–1.55	–1.28	–0.22	–0.03	–0.01
	5	–1.88	–1.56	–0.27	–0.04	–0.01
	6	–2.10	–1.75	–0.30	–0.04	–0.01
	7	–2.26	–1.88	–0.32	–0.05	–0.01
	8	–2.38	–1.98	–0.34	–0.05	–0.01
	9	–2.47	–2.06	–0.35	–0.05	–0.01
	10	–2.54	–2.12	–0.36	–0.05	–0.01
	11	–2.60	–2.17	–0.37	–0.05	–0.01
	12	–2.65	–2.21	–0.37	–0.05	–0.01

[a] Computed at ZORA-BLYP-D3(BJ)/TZ2P in $C_{\infty v}$ symmetry.

Table S4. Energy decomposition analysis of $\Delta\bar{E}_{syn}$ [in kcal mol^{-1}] of (X–CC–CN)$_n$ with X = H, Cl, Br, and I.[a]

System	n	$\Delta\bar{E}_{syn}$	$\Delta E_{syn,oi}$	$\Delta V_{syn,elstat}$	$\Delta E_{syn,Pauli}$	$\Delta E_{syn,disp}$
**H–CC–CN**	3	–0.38	–0.28	–0.11	0.00	–0.01
	4	–0.57	–0.42	–0.16	0.01	0.00
	5	–0.69	–0.50	–0.19	0.00	–0.01
	6	–0.76	–0.56	–0.21	0.01	0.00
	7	–0.82	–0.59	–0.23	0.01	0.00
	8	–0.86	–0.62	–0.24	0.01	0.00
	9	–0.89	–0.65	–0.25	0.01	0.00
	10	–0.91	–0.66	–0.25	0.01	0.00
	11	–0.93	–0.68	–0.26	0.01	0.00
	12	–0.95	–0.69	–0.26	0.01	0.00
**Cl–CC–CN**	3	–0.29	–0.21	–0.07	0.00	–0.01
	4	–0.44	–0.32	–0.11	0.00	0.00
	5	–0.52	–0.39	–0.13	0.00	–0.01
	6	–0.58	–0.43	–0.15	0.00	0.00
	7	–0.62	–0.46	–0.16	0.00	–0.01
	8	–0.65	–0.48	–0.17	0.00	0.00
	9	–0.68	–0.49	–0.18	0.00	0.00
	10	–0.70	–0.51	–0.18	0.00	0.00
	11	–0.71	–0.52	–0.19	0.00	0.00
	12	–0.73	–0.53	–0.19	0.00	0.00
**Br–CC–CN**	3	–0.50	–0.37	–0.09	0.00	0.00
	4	–0.73	–0.56	–0.14	0.00	0.00
	5	–0.88	–0.68	–0.16	0.00	0.00
	6	–0.97	–0.75	–0.19	0.00	0.00
	7	–1.04	–0.81	–0.20	0.00	0.00
	8	–1.09	–0.84	–0.21	0.00	0.00
	9	–1.13	–0.87	–0.22	0.00	0.00
	10	–1.16	–0.90	–0.23	0.00	0.00
	11	–1.18	–0.92	–0.23	0.00	0.00
	12	–1.20	–0.93	–0.24	0.00	0.00
**I–CC–CN**	3	–0.65	–0.54	–0.10	–0.01	0.00
	4	–0.99	–0.82	–0.15	–0.01	0.00
	5	–1.20	–0.99	–0.19	–0.01	0.00
	6	–1.33	–1.10	–0.21	–0.01	0.00
	7	–1.43	–1.18	–0.23	–0.01	0.00
	8	–1.50	–1.24	–0.24	–0.01	0.00
	9	–1.55	–1.28	–0.25	–0.01	0.00
	10	–1.60	–1.32	–0.26	–0.01	0.00
	11	–1.63	–1.35	–0.27	–0.01	0.00
	12	–1.66	–1.37	–0.27	–0.01	0.00

[a] Computed at ZORA-BLYP-D3(BJ)/TZ2P in $C_{\infty v}$ symmetry.

Table S5. Energy decomposition analysis of $\Delta\bar{E}_{syn}$ [in kcal mol^{-1}] of $(X–C_6H_4–CN)_n$ with X = H, Cl, Br, and I.[a]

System	n	$\Delta\bar{E}_{syn}$	$\Delta E_{syn,oi}$	$\Delta V_{syn,elstat}$	$\Delta E_{syn,Pauli}$	$\Delta E_{syn,disp}$
**$H–C_6H_4–CN$**	3	–0.08	–0.04	–0.04	0.00	0.00
	4	–0.11	–0.06	–0.06	0.00	0.00
	5	–0.13	–0.07	–0.07	0.00	0.00
	6	–0.14	–0.07	–0.07	0.00	0.00
	7	–0.15	–0.08	–0.08	0.00	0.00
	8	–0.16	–0.08	–0.08	0.00	0.00
	9	–0.17	–0.08	–0.08	0.00	0.00
	10	–0.17	–0.08	–0.09	0.00	0.00
	11	–0.17	–0.09	–0.09	0.00	0.00
	12	–0.17	–0.09	–0.09	0.00	0.00
**$Cl–C_6H_4–CN$**	3	–0.06	–0.06	–0.02	0.00	0.00
	4	–0.10	–0.08	–0.03	0.00	0.00
	5	–0.12	–0.10	–0.03	0.00	0.00
	6	–0.13	–0.10	–0.03	0.00	0.00
	7	–0.14	–0.11	–0.04	0.00	0.00
	8	–0.15	–0.12	–0.04	0.00	0.00
	9	–0.15	–0.12	–0.04	0.00	0.00
	10	–0.16	–0.12	–0.04	0.00	0.00
	11	–0.16	–0.13	–0.04	0.00	0.00
	12	–0.16	–0.13	–0.04	0.00	0.00
**$Br–C_6H_4–CN$**	3	–0.10	–0.09	–0.01	0.00	0.00
	4	–0.15	–0.13	–0.02	0.00	0.00
	5	–0.18	–0.16	–0.03	0.00	0.00
	6	–0.20	–0.17	–0.03	0.00	0.00
	7	–0.21	–0.18	–0.03	0.00	0.00
	8	–0.22	–0.19	–0.03	0.00	0.00
	9	–0.23	–0.20	–0.04	0.00	0.00
	10	–0.23	–0.20	–0.04	0.00	0.00
	11	–0.24	–0.21	–0.04	0.00	0.00
	12	–0.24	–0.21	–0.04	0.00	0.00
**$I–C_6H_4–CN$**	3	–0.16	–0.14	–0.02	0.00	0.00
	4	–0.24	–0.21	–0.03	–0.01	0.00
	5	–0.29	–0.25	–0.04	–0.01	0.00
	6	–0.32	–0.27	–0.04	–0.01	0.00
	7	–0.34	–0.29	–0.04	–0.01	0.00
	8	–0.35	–0.30	–0.05	–0.01	0.00
	9	–0.37	–0.31	–0.05	–0.01	0.00
	10	–0.38	–0.32	–0.05	–0.01	0.00
	11	–0.38	–0.33	–0.05	–0.01	0.00
	12	–0.39	–0.33	–0.05	–0.01	0.00

[a] Computed at ZORA-BLYP-D3(BJ)/TZ2P in C_S symmetry.

Table S6. Energy decomposition [in kcal mol^{-1}] of the ΔE_{int} between the chain of n units and an additional monomer for $(X–CN)_{n+1}$ with X = H, Cl, Br, and I, where the additional monomer is added at the hydrogen or halogen bond acceptor side of the chain.[a]

System	n	ΔE_{Pauli}	ΔV_{elstat}	ΔE_{oi}	ΔE_{σ}	ΔE_{π}	ΔE_{disp}	ΔE_{int}
**H–CN**	1 + 1	8.9	–8.0	–4.3	–3.9	–0.5	–1.1	–4.3
	2 + 1	9.1	–9.5	–4.7	–4.2	–0.6	–1.1	–6.1
	3 + 1	9.2	–9.9	–4.8	–4.2	–0.6	–1.1	–6.6
	4 + 1	9.2	–10.0	–4.9	–4.3	–0.6	–1.1	–6.7
	5 + 1	9.2	–10.1	–4.9	–4.3	–0.6	–1.1	–6.8
	6 + 1	9.2	–10.1	–4.9	–4.3	–0.6	–1.1	–6.9
	7 + 1	9.2	–10.2	–4.9	–4.3	–0.6	–1.1	–6.9
	8 + 1	9.2	–10.2	–4.9	–4.3	–0.6	–1.1	–6.9
	9 + 1	9.2	–10.2	–4.9	–4.3	–0.6	–1.1	–6.9
	10 + 1	9.2	–10.2	–4.9	–4.3	–0.6	–1.1	–6.9
	11 + 1	9.2	–10.2	–4.9	–4.3	–0.6	–1.1	–7.0
**Cl–CN**	1 + 1	5.8	–5.4	–2.2	–1.8	–0.4	–1.3	–3.1
	2 + 1	5.9	–6.0	–2.4	–2.0	–0.5	–1.3	–3.8
	3 + 1	5.9	–6.2	–2.5	–2.0	–0.5	–1.3	–4.0
	4 + 1	5.9	–6.3	–2.5	–2.0	–0.5	–1.3	–4.1
	5 + 1	6.0	–6.3	–2.5	–2.0	–0.5	–1.3	–4.2
	6 + 1	6.0	–6.3	–2.5	–2.0	–0.5	–1.3	–4.2
	7 + 1	6.0	–6.3	–2.5	–2.0	–0.5	–1.3	–4.2
	8 + 1	6.0	–6.3	–2.5	–2.0	–0.5	–1.3	–4.2
	9 + 1	6.0	–6.3	–2.5	–2.0	–0.5	–1.3	–4.2
	10 + 1	6.0	–6.3	–2.5	–2.0	–0.5	–1.3	–4.2
	11 + 1	6.0	–6.3	–2.5	–2.0	–0.5	–1.3	–4.2
**Br–CN**	1 + 1	11.8	–9.5	–5.3	–4.4	–0.9	–1.8	–4.8
	2 + 1	12.2	–10.7	–5.8	–4.7	–1.0	–1.8	–6.1
	3 + 1	12.3	–11.1	–5.9	–4.9	–1.0	–1.8	–6.5
	4 + 1	12.3	–11.2	–5.9	–4.9	–1.0	–1.8	–6.7
	5 + 1	12.3	–11.3	–6.0	–4.9	–1.1	–1.8	–6.7
	6 + 1	12.3	–11.3	–6.0	–4.9	–1.1	–1.8	–6.8
	7 + 1	12.3	–11.3	–6.0	–4.9	–1.1	–1.8	–6.8
	8 + 1	12.3	–11.3	–6.0	–4.9	–1.1	–1.8	–6.8
	9 + 1	12.3	–11.3	–6.0	–4.9	–1.1	–1.8	–6.8
	10 + 1	12.3	–11.3	–6.0	–4.9	–1.1	–1.8	–6.8
	11 + 1	12.3	–11.3	–6.0	–4.9	–1.1	–1.8	–6.8
**I–CN**	1 + 1	15.9	–12.8	–7.5	–6.0	–1.5	–2.3	–6.6
	2 + 1	16.5	–14.6	–8.2	–6.7	–1.6	–2.3	–8.5
	3 + 1	16.6	–15.2	–8.5	–6.9	–1.6	–2.3	–9.3
	4 + 1	16.6	–15.4	–8.5	–6.9	–1.6	–2.3	–9.5
	5 + 1	16.6	–15.4	–8.6	–7.0	–1.6	–2.3	–9.6
	6 + 1	16.7	–15.5	–8.6	–7.0	–1.6	–2.3	–9.7
	7 + 1	16.7	–15.5	–8.6	–7.0	–1.6	–2.3	–9.7
	8 + 1	16.7	–15.5	–8.6	–7.0	–1.6	–2.3	–9.8
	9 + 1	16.7	–15.5	–8.6	–7.0	–1.6	–2.3	–9.8
	10 + 1	16.7	–15.5	–8.6	–7.0	–1.6	–2.3	–9.8
	11 + 1	16.7	–15.5	–8.6	–7.0	–1.6	–2.3	–9.8

[a] Energies computed at ZORA-BLYP-D3(BJ)/TZ2P in $C_{\infty v}$ symmetry.

Table S7. Energy decomposition [in kcal mol^{-1}] of the ΔE_{int} between the chain of n units and an additional monomer for $(X–CC–CN)_{n+1}$ with X = H, Cl, Br, and I, where the additional monomer is added at the hydrogen or halogen bond acceptor side of the chain.[a]

System	n	$\Delta E_{\mathrm{Pauli}}$	$\Delta V_{\mathrm{elstat}}$	ΔE_{oi}	ΔE_{σ}	ΔE_{π}	ΔE_{disp}	ΔE_{int}
**H–CC–CN**	1 + 1	4.7	–5.6	–2.7	–2.2	–0.5	–1.0	–4.6
	2 + 1	4.8	–6.3	–2.9	–2.3	–0.5	–1.0	–5.3
	3 + 1	4.8	–6.5	–2.9	–2.3	–0.6	–1.0	–5.5
	4 + 1	4.8	–6.5	–2.9	–2.3	–0.6	–1.0	–5.6
	5 + 1	4.8	–6.5	–2.9	–2.3	–0.6	–1.0	–5.6
	6 + 1	4.8	–6.6	–2.9	–2.4	–0.6	–1.0	–5.7
	7 + 1	4.8	–6.6	–2.9	–2.4	–0.6	–1.0	–5.7
	8 + 1	4.8	–6.6	–2.9	–2.4	–0.6	–1.0	–5.7
	9 + 1	4.8	–6.6	–2.9	–2.4	–0.6	–1.0	–5.7
	10 + 1	4.8	–6.6	–2.9	–2.4	–0.6	–1.0	–5.7
	11 + 1	4.8	–6.6	–2.9	–2.4	–0.6	–1.0	–5.7
**Cl–CC–CN**	1 + 1	5.1	–5.3	–2.1	–1.6	–0.5	–1.2	–3.6
	2 + 1	5.2	–5.8	–2.2	–1.7	–0.6	–1.2	–4.1
	3 + 1	5.2	–5.9	–2.3	–1.7	–0.6	–1.2	–4.3
	4 + 1	5.2	–6.0	–2.3	–1.7	–0.6	–1.2	–4.3
	5 + 1	5.2	–6.0	–2.3	–1.7	–0.6	–1.2	–4.4
	6 + 1	5.2	–6.0	–2.3	–1.7	–0.6	–1.2	–4.4
	7 + 1	5.2	–6.0	–2.3	–1.7	–0.6	–1.2	–4.4
	8 + 1	5.2	–6.0	–2.3	–1.7	–0.6	–1.2	–4.4
	9 + 1	5.2	–6.0	–2.3	–1.7	–0.6	–1.2	–4.4
	10 + 1	5.2	–6.1	–2.3	–1.7	–0.6	–1.2	–4.4
	11 + 1	5.2	–6.1	–2.3	–1.7	–0.6	–1.2	–4.4
**Br–CC–CN**	1 + 1	9.5	–8.7	–4.5	–3.5	–1.0	–1.7	–5.4
	2 + 1	9.7	–9.5	–4.8	–3.7	–1.1	–1.7	–6.3
	3 + 1	9.7	–9.7	–4.8	–3.7	–1.1	–1.7	–6.5
	4 + 1	9.7	–9.8	–4.9	–3.7	–1.1	–1.7	–6.6
	5 + 1	9.7	–9.9	–4.9	–3.7	–1.1	–1.7	–6.7
	6 + 1	9.7	–9.9	–4.9	–3.7	–1.1	–1.7	–6.7
	7 + 1	9.7	–9.9	–4.9	–3.7	–1.1	–1.7	–6.7
	8 + 1	9.7	–9.9	–4.9	–3.8	–1.1	–1.7	–6.7
	9 + 1	9.7	–9.9	–4.9	–3.8	–1.1	–1.7	–6.7
	10 + 1	9.7	–9.9	–4.9	–3.8	–1.1	–1.7	–6.7
	11 + 1	9.7	–9.9	–4.9	–3.8	–1.1	–1.7	–6.7
**I–CC–CN**	1 + 1	13.2	–11.8	–6.3	–4.8	–1.6	–2.1	–7.0
	2 + 1	13.4	–12.9	–6.8	–5.1	–1.7	–2.1	–8.3
	3 + 1	13.5	–13.2	–6.9	–5.2	–1.7	–2.1	–8.7
	4 + 1	13.5	–13.3	–6.9	–5.2	–1.7	–2.1	–8.8
	5 + 1	13.5	–13.3	–6.9	–5.2	–1.7	–2.1	–8.9
	6 + 1	13.5	–13.4	–6.9	–5.2	–1.7	–2.1	–8.9
	7 + 1	13.5	–13.4	–6.9	–5.2	–1.7	–2.1	–8.9
	8 + 1	13.5	–13.4	–6.9	–5.2	–1.7	–2.1	–8.9
	9 + 1	13.5	–13.4	–6.9	–5.2	–1.7	–2.1	–8.9
	10 + 1	13.5	–13.4	–6.9	–5.2	–1.7	–2.1	–9.0
	11 + 1	13.5	–13.4	–6.9	–5.2	–1.7	–2.1	–9.0

[a] Energies computed at ZORA-BLYP-D3(BJ)/TZ2P in $C_{\infty v}$ symmetry.

Table S8. Energy decomposition [in kcal mol^{-1}] of the ΔE_{int} between the chain of n units and an additional monomer for $(X–C_6H_4–CN)_{n+1}$ with X = H, Cl, Br, and I, where the additional monomer is added at the hydrogen or halogen bond acceptor side of the chain.[a]

System	n	ΔE_{Pauli}	ΔV_{elstat}	ΔE_{oi}	ΔE_{σ}	ΔE_{π}	ΔE_{disp}	ΔE_{int}
**$H–C_6H_4–CN$**	1 + 1	0.2	–1.2	–0.2	–0.1	–0.1	–0.3	–1.5
	2 + 1	0.2	–1.3	–0.2	–0.1	–0.1	–0.3	–1.6
	3 + 1	0.2	–1.3	–0.2	–0.1	–0.1	–0.3	–1.6
	4 + 1	0.2	–1.3	–0.2	–0.1	–0.1	–0.3	–1.6
	5 + 1	0.2	–1.3	–0.2	–0.1	–0.1	–0.3	–1.7
	6 + 1	0.2	–1.3	–0.2	–0.1	–0.1	–0.3	–1.7
	7 + 1	0.2	–1.3	–0.2	–0.1	–0.1	–0.3	–1.7
	8 + 1	0.2	–1.3	–0.2	–0.1	–0.1	–0.3	–1.7
	9 + 1	0.2	–1.3	–0.2	–0.1	–0.1	–0.3	–1.7
	10 + 1	0.2	–1.3	–0.2	–0.1	–0.1	–0.3	–1.7
	11 + 1	0.2	–1.3	–0.2	–0.1	–0.1	–0.3	–1.7
**$Cl–C_6H_4–CN$**	1 + 1	3.0	–1.9	–1.1	–0.9	–0.2	–1.1	–1.2
	2 + 1	3.0	–2.0	–1.2	–1.0	–0.3	–1.1	–1.4
	3 + 1	3.0	–2.0	–1.2	–1.0	–0.3	–1.1	–1.4
	4 + 1	3.0	–2.1	–1.2	–1.0	–0.3	–1.1	–1.4
	5 + 1	3.0	–2.1	–1.2	–1.0	–0.3	–1.1	–1.4
	6 + 1	3.0	–2.1	–1.2	–1.0	–0.3	–1.1	–1.4
	7 + 1	3.0	–2.1	–1.2	–1.0	–0.3	–1.1	–1.4
	8 + 1	3.0	–2.1	–1.2	–1.0	–0.3	–1.1	–1.4
	9 + 1	3.0	–2.1	–1.2	–1.0	–0.3	–1.1	–1.4
	10 + 1	3.0	–2.1	–1.2	–1.0	–0.3	–1.1	–1.4
	11 + 1	3.0	–2.1	–1.2	–1.0	–0.3	–1.1	–1.4
**$Br–C_6H_4–CN$**	1 + 1	4.2	–3.3	–1.9	–1.7	–0.2	–1.4	–2.4
	2 + 1	4.2	–3.4	–2.0	–1.8	–0.2	–1.4	–2.6
	3 + 1	4.2	–3.4	–2.0	–1.8	–0.2	–1.4	–2.7
	4 + 1	4.2	–3.4	–2.0	–1.8	–0.2	–1.4	–2.7
	5 + 1	4.2	–3.4	–2.0	–1.8	–0.2	–1.4	–2.7
	6 + 1	4.2	–3.4	–2.0	–1.8	–0.2	–1.4	–2.7
	7 + 1	4.2	–3.4	–2.0	–1.8	–0.2	–1.4	–2.7
	8 + 1	4.2	–3.5	–2.0	–1.8	–0.2	–1.4	–2.7
	9 + 1	4.2	–3.5	–2.0	–1.8	–0.2	–1.4	–2.7
	10 + 1	4.2	–3.5	–2.0	–1.8	–0.2	–1.4	–2.7
	11 + 1	4.2	–3.5	–2.0	–1.8	–0.2	–1.4	–2.7
**$I–C_6H_4–CN$**	1 + 1	6.3	–5.3	–2.8	–2.5	–0.3	–1.9	–3.6
	2 + 1	6.4	–5.5	–2.9	–2.6	–0.3	–1.9	–4.0
	3 + 1	6.4	–5.6	–3.0	–2.6	–0.3	–1.9	–4.0
	4 + 1	6.4	–5.6	–3.0	–2.6	–0.4	–1.9	–4.1
	5 + 1	6.4	–5.6	–3.0	–2.6	–0.4	–1.9	–4.1
	6 + 1	6.4	–5.6	–3.0	–2.6	–0.4	–1.9	–4.1
	7 + 1	6.4	–5.6	–3.0	–2.6	–0.4	–1.9	–4.1
	8 + 1	6.4	–5.6	–3.0	–2.6	–0.4	–1.9	–4.1
	9 + 1	6.4	–5.6	–3.0	–2.7	–0.4	–1.9	–4.1
	10 + 1	6.4	–5.6	–3.0	–2.7	–0.4	–1.9	–4.1
	11 + 1	6.4	–5.6	–3.0	–2.7	–0.4	–1.9	–4.1

[a] Energies computed at ZORA-BLYP-D3(BJ)/TZ2P in C_S symmetry.

Table S9. Energy decomposition [in kcal mol^{-1}] of the ΔE_{int} between the chain of n units and an additional monomer for $(X–CN)_{n+1}$ with X = H, Cl, Br, and I, where the additional monomer is added at the hydrogen or halogen bond donor side of the chain.[a]

System	n	ΔE_{Pauli}	ΔV_{elstat}	ΔE_{oi}	ΔE_{σ}	ΔE_{π}	ΔE_{disp}	ΔE_{int}
**H–CN**	1 + 1	8.9	–8.0	–4.3	–3.9	–0.5	–1.1	–4.3
	2 + 1	8.7	–9.0	–4.7	–4.1	–0.6	–1.1	–6.1
	3 + 1	8.7	–9.3	–4.8	–4.2	–0.6	–1.1	–6.6
	4 + 1	8.6	–9.4	–4.9	–4.2	–0.7	–1.1	–6.7
	5 + 1	8.6	–9.5	–4.9	–4.3	–0.7	–1.1	–6.8
	6 + 1	8.6	–9.5	–4.9	–4.3	–0.7	–1.1	–6.9
	7 + 1	8.6	–9.5	–4.9	–4.3	–0.7	–1.1	–6.9
	8 + 1	8.6	–9.6	–4.9	–4.3	–0.7	–1.1	–6.9
	9 + 1	8.6	–9.6	–4.9	–4.3	–0.7	–1.1	–6.9
	10 + 1	8.6	–9.6	–4.9	–4.3	–0.7	–1.1	–6.9
	11 + 1	8.6	–9.6	–4.9	–4.3	–0.7	–1.1	–7.0
**Cl–CN**	1 + 1	5.8	–5.4	–2.2	–1.8	–0.4	–1.3	–3.1
	2 + 1	5.7	–6.0	–2.3	–1.9	–0.4	–1.3	–3.8
	3 + 1	5.7	–6.1	–2.4	–1.9	–0.5	–1.3	–4.0
	4 + 1	5.7	–6.2	–2.4	–1.9	–0.5	–1.3	–4.1
	5 + 1	5.7	–6.2	–2.4	–1.9	–0.5	–1.3	–4.2
	6 + 1	5.7	–6.2	–2.4	–1.9	–0.5	–1.3	–4.2
	7 + 1	5.7	–6.2	–2.4	–1.9	–0.5	–1.3	–4.2
	8 + 1	5.7	–6.2	–2.4	–1.9	–0.5	–1.3	–4.2
	9 + 1	5.7	–6.2	–2.4	–1.9	–0.5	–1.3	–4.2
	10 + 1	5.7	–6.2	–2.4	–1.9	–0.5	–1.3	–4.2
	11 + 1	5.7	–6.2	–2.4	–1.9	–0.5	–1.3	–4.2
**Br–CN**	1 + 1	11.8	–9.5	–5.3	–4.4	–0.9	–1.8	–4.8
	2 + 1	11.7	–10.5	–5.5	–4.6	–1.0	–1.8	–6.1
	3 + 1	11.6	–10.7	–5.7	–4.6	–1.0	–1.8	–6.5
	4 + 1	11.6	–10.8	–5.7	–4.7	–1.0	–1.8	–6.7
	5 + 1	11.6	–10.9	–5.7	–4.7	–1.0	–1.8	–6.8
	6 + 1	11.6	–10.9	–5.7	–4.7	–1.1	–1.8	–6.8
	7 + 1	11.6	–10.9	–5.8	–4.7	–1.1	–1.8	–6.8
	8 + 1	11.6	–10.9	–5.8	–4.7	–1.1	–1.8	–6.8
	9 + 1	11.6	–10.9	–5.8	–4.7	–1.1	–1.8	–6.8
	10 + 1	11.6	–10.9	–5.8	–4.7	–1.1	–1.8	–6.8
	11 + 1	11.6	–10.9	–5.8	–4.7	–1.1	–1.8	–6.9
**I–CN**	1 + 1	15.9	–12.8	–7.5	–6.0	–1.5	–2.3	–6.6
	2 + 1	15.6	–14.0	–8.1	–6.5	–1.6	–2.3	–8.7
	3 + 1	15.5	–14.3	–8.3	–6.6	–1.7	–2.3	–9.3
	4 + 1	15.5	–14.4	–8.4	–6.7	–1.7	–2.3	–9.6
	5 + 1	15.5	–14.5	–8.4	–6.7	–1.7	–2.3	–9.7
	6 + 1	15.5	–14.5	–8.4	–6.7	–1.7	–2.3	–9.7
	7 + 1	15.5	–14.5	–8.4	–6.7	–1.7	–2.3	–9.7
	8 + 1	15.5	–14.6	–8.5	–6.7	–1.8	–2.3	–9.8
	9 + 1	15.5	–14.6	–8.5	–6.7	–1.8	–2.3	–9.8
	10 + 1	15.5	–14.6	–8.5	–6.7	–1.8	–2.3	–9.8
	11 + 1	15.5	–14.6	–8.5	–6.7	–1.8	–2.3	–9.8

[a] Energies computed at ZORA-BLYP-D3(BJ)/TZ2P in $C_{\infty v}$ symmetry.

Table S10. Energy decomposition [in kcal mol^{-1}] of the ΔE_{int} between the chain of n units and an additional monomer for $(X\text{–}CC\text{–}CN)_{n+1}$ with X = H, Cl, Br, and I, where the additional monomer is added at the hydrogen or halogen bond donor side of the chain.[a]

System	n	ΔE_{Pauli}	ΔV_{elstat}	ΔE_{oi}	ΔE_{σ}	ΔE_{π}	ΔE_{disp}	ΔE_{int}
**H–CC–CN**	1 + 1	4.7	–5.6	–2.7	–2.2	–0.5	–1.0	–4.6
	2 + 1	4.7	–6.2	–2.9	–2.3	–0.6	–1.0	–5.3
	3 + 1	4.7	–6.3	–2.9	–2.3	–0.6	–1.0	–5.5
	4 + 1	4.7	–6.4	–2.9	–2.3	–0.6	–1.0	–5.6
	5 + 1	4.7	–6.4	–2.9	–2.3	–0.6	–1.0	–5.6
	6 + 1	4.7	–6.4	–2.9	–2.3	–0.6	–1.0	–5.7
	7 + 1	4.7	–6.4	–2.9	–2.3	–0.6	–1.0	–5.7
	8 + 1	4.7	–6.4	–2.9	–2.3	–0.6	–1.0	–5.7
	9 + 1	4.7	–6.4	–2.9	–2.3	–0.6	–1.0	–5.7
	10 + 1	4.7	–6.4	–2.9	–2.3	–0.6	–1.0	–5.7
	11 + 1	4.7	–6.4	–2.9	–2.3	–0.6	–1.0	–5.7
**Cl–CC–CN**	1 + 1	5.1	–5.3	–2.1	–1.6	–0.5	–1.2	–3.6
	2 + 1	5.1	–5.8	–2.2	–1.6	–0.6	–1.2	–4.1
	3 + 1	5.1	–5.9	–2.2	–1.6	–0.6	–1.2	–4.3
	4 + 1	5.1	–5.9	–2.2	–1.6	–0.6	–1.2	–4.3
	5 + 1	5.1	–6.0	–2.2	–1.6	–0.6	–1.2	–4.4
	6 + 1	5.1	–6.0	–2.2	–1.6	–0.6	–1.2	–4.4
	7 + 1	5.1	–6.0	–2.2	–1.6	–0.6	–1.2	–4.4
	8 + 1	5.1	–6.0	–2.2	–1.6	–1.6	–1.2	–4.4
	9 + 1	5.1	–6.0	–2.2	–1.6	–0.6	–1.2	–4.4
	10 + 1	5.1	–6.0	–2.2	–1.6	–0.6	–1.2	–4.4
	11 + 1	5.1	–6.0	–2.2	–1.6	–0.6	–1.2	–4.4
**Br–CC–CN**	1 + 1	9.5	–8.7	–4.5	–3.5	–1.0	–1.7	–5.4
	2 + 1	9.5	–9.3	–4.7	–3.6	–1.0	–1.7	–6.3
	3 + 1	9.4	–9.5	–4.8	–3.6	–1.1	–1.7	–6.5
	4 + 1	9.4	–9.6	–4.8	–3.7	–1.2	–1.7	–6.6
	5 + 1	9.4	–9.6	–4.8	–3.7	–1.2	–1.7	–6.7
	6 + 1	9.4	–9.7	–4.8	–3.7	–1.2	–1.7	–6.7
	7 + 1	9.4	–9.7	–4.8	–3.7	–1.2	–1.7	–6.7
	8 + 1	9.4	–9.7	–4.8	–3.7	–1.2	–1.7	–6.7
	9 + 1	9.4	–9.7	–4.8	–3.7	–1.2	–1.7	–6.7
	10 + 1	9.4	–9.7	–4.8	–3.7	–1.2	–1.7	–6.7
	11 + 1	9.4	–9.7	–4.8	–3.7	–1.2	–1.7	–6.7
**I–CC–CN**	1 + 1	13.2	–11.8	–6.3	–4.8	–1.6	–2.1	–7.0
	2 + 1	13.1	–12.6	–6.7	–5.0	–1.7	–2.1	–8.3
	3 + 1	13.1	–12.8	–6.8	–5.0	–1.8	–2.1	–8.7
	4 + 1	13.1	–12.9	–6.9	–5.1	–1.8	–2.1	–8.8
	5 + 1	13.1	–12.9	–6.9	–5.1	–1.8	–2.1	–8.9
	6 + 1	13.1	–12.9	–6.9	–5.1	–1.8	–2.1	–8.9
	7 + 1	13.1	–13.0	–6.9	–5.1	–1.8	–2.1	–8.9
	8 + 1	13.1	–13.0	–6.9	–5.1	–1.8	–2.1	–8.9
	9 + 1	13.1	–13.0	–6.9	–5.1	–1.8	–2.1	–8.9
	10 + 1	13.1	–13.0	–6.9	–5.1	–1.8	–2.1	–9.0
	11 + 1	13.1	–13.0	–6.9	–5.1	–1.8	–2.1	–9.0

[a] Energies computed at ZORA-BLYP-D3(BJ)/TZ2P in $C_{\infty v}$ symmetry.

Table S11. Energy decomposition [in kcal mol^{-1}] of the ΔE_{int} between the chain of n units and an additional monomer for $(X–C_6H_4–CN)_{n+1}$ with X = H, Cl, Br, and I where the additional monomer is added at the hydrogen or halogen bond donor side of the chain.[a]

System	n	ΔE_{Pauli}	ΔV_{elstat}	ΔE_{oi}	ΔE_{σ}	ΔE_{π}	ΔE_{disp}	ΔE_{int}
**$H–C_6H_4–CN$**	1 + 1	0.2	–1.2	–0.2	–0.1	–0.1	–0.3	–1.5
	2 + 1	0.2	–1.3	–0.2	–0.1	–0.1	–0.3	–1.6
	3 + 1	0.2	–1.3	–0.2	–0.1	–0.1	–0.3	–1.6
	4 + 1	0.2	–1.3	–0.2	–0.1	–0.1	–0.3	–1.6
	5 + 1	0.2	–1.3	–0.2	–0.1	–0.1	–0.3	–1.7
	6 + 1	0.2	–1.3	–0.2	–0.1	–0.1	–0.3	–1.7
	7 + 1	0.2	–1.3	–0.2	–0.1	–0.1	–0.3	–1.7
	8 + 1	0.2	–1.3	–0.2	–0.1	–0.1	–0.3	–1.7
	9 + 1	0.2	–1.3	–0.2	–0.1	–0.1	–0.3	–1.7
	10 + 1	0.2	–1.3	–0.2	–0.1	–0.1	–0.3	–1.7
	11 + 1	0.2	–1.3	–0.2	–0.1	–0.1	–0.3	–1.7
**$Cl–C_6H_4–CN$**	1 + 1	3.0	–1.9	–1.1	–0.9	–0.2	–1.1	–1.2
	2 + 1	3.0	–2.1	–1.1	–0.9	–0.2	–1.1	–1.3
	3 + 1	3.0	–2.1	–1.1	–0.9	–0.2	–1.1	–1.3
	4 + 1	3.0	–2.1	–1.1	–0.9	–0.2	–1.1	–1.3
	5 + 1	3.0	–2.1	–1.1	–0.9	–0.2	–1.1	–1.3
	6 + 1	3.0	–2.1	–1.1	–0.9	–0.2	–1.1	–1.3
	7 + 1	3.0	–2.1	–1.1	–0.9	–0.2	–1.1	–1.3
	8 + 1	3.0	–2.1	–1.1	–0.9	–0.2	–1.1	–1.3
	9 + 1	3.0	–2.1	–1.1	–0.9	–0.2	–1.1	–1.3
	10 + 1	3.0	–2.1	–1.1	–0.9	–0.2	–1.1	–1.3
	11 + 1	3.0	–2.1	–1.1	–0.9	–0.2	–1.1	–1.3
**$Br–C_6H_4–CN$**	1 + 1	4.2	–3.3	–1.9	–1.7	–0.2	–1.4	–2.4
	2 + 1	4.2	–3.5	–1.8	–1.6	–0.2	–1.4	–2.6
	3 + 1	4.2	–3.5	–1.8	–1.6	–0.2	–1.4	–2.6
	4 + 1	4.2	–3.5	–1.8	–1.6	–0.2	–1.4	–2.6
	5 + 1	4.2	–3.5	–1.8	–1.6	–0.2	–1.4	–2.6
	6 + 1	4.2	–3.5	–1.8	–1.6	–0.2	–1.4	–2.6
	7 + 1	4.2	–3.5	–1.8	–1.6	–0.2	–1.4	–2.6
	8 + 1	4.2	–3.5	–1.8	–1.6	–0.2	–1.4	–2.6
	9 + 1	4.2	–3.5	–1.8	–1.6	–0.2	–1.4	–2.6
	10 + 1	4.2	–3.5	–1.8	–1.6	–0.2	–1.4	–2.6
	11 + 1	4.2	–3.5	–1.8	–1.6	–0.2	–1.4	–2.6
**$I–C_6H_4–CN$**	1 + 1	6.3	–5.3	–2.8	–2.5	–0.3	–1.9	–3.6
	2 + 1	6.3	–5.6	–2.8	–2.5	–0.3	–1.9	–4.0
	3 + 1	6.3	–5.6	–2.8	–2.5	–0.3	–1.9	–4.0
	4 + 1	6.3	–5.7	–2.8	–2.5	–0.3	–1.9	–4.0
	5 + 1	6.3	–5.7	–2.8	–2.5	–0.3	–1.9	–4.0
	6 + 1	6.3	–5.7	–2.8	–2.5	–0.3	–1.9	–4.0
	7 + 1	6.3	–5.7	–2.8	–2.5	–0.3	–1.9	–4.0
	8 + 1	6.3	–5.7	–2.8	–2.5	–0.3	–1.9	–4.0
	9 + 1	6.3	–5.7	–2.8	–2.5	–0.3	–1.9	–4.0
	10 + 1	6.3	–5.7	–2.8	–2.5	–0.3	–1.9	–4.0
	11 + 1	6.3	–5.7	–2.8	–2.5	–0.3	–1.9	–4.0

[a] Energies computed at ZORA-BLYP-D3(BJ)/TZ2P in C_S symmetry.

Table S12. The VDD charges [in milli-electrons] of the first and last monomers in the $(X–CN)_n$, $(X–CC–CN)_n$ and $(X–C_6H_4–CN)_n$ systems, with X = H, Cl, Br, and I.[a]

	**n**	**H**		**Cl**		**Br**		**I**	
		First	Last	First	Last	First	Last	First	Last
**X–CN**	2	74	–74	38	–39	77	–77	119	–119
	3	82	–82	41	–41	84	–84	130	–130
	4	83	–83	42	–42	85	–85	133	–133
	5	83	–83	42	–42	86	–86	133	–133
	6	83	–83	42	–42	86	–86	133	–133
	7	83	–83	42	–42	86	–86	133	–133
	8	83	–83	42	–42	86	–86	133	–133
	9	83	–83	42	–42	86	–86	133	–133
	10	83	–83	42	–42	86	–86	133	–133
	11	83	–83	42	–42	86	–86	133	–133
	12	83	–83	42	–42	86	–86	133	–133
**X–CC–CN**	2	55	–55	36	–36	70	–70	106	–106
	3	58	–58	38	–38	75	–75	114	–114
	4	58	–58	38	–38	75	–75	116	–116
	5	58	–58	38	–38	75	–75	116	–116
	6	58	–58	38	–38	75	–75	116	–116
	7	58	–58	38	–38	75	–75	116	–116
	8	58	–58	38	–38	75	–75	116	–116
	9	58	–58	38	–38	75	–75	116	–116
	10	58	–58	38	–38	75	–75	116	–116
	11	58	–58	38	–38	75	–75	116	–116
	12	58	–58	38	–38	75	–75	116	–116
**X–C$_6$H$_4$–CN**	2	7	–7	20	–20	36	–36	63	–63
	3	7	–7	20	–20	36	–36	63	–63
	4	7	–7	20	–20	36	–36	63	–63
	5	7	–7	20	–20	36	–36	63	–63
	6	7	–7	20	–20	36	–36	63	–63
	7	7	–7	20	–20	36	–36	63	–63
	8	7	–7	20	–20	36	–36	63	–63
	9	7	–7	20	–20	36	–36	63	–63
	10	7	–7	20	–20	36	–36	63	–63
	11	7	–7	20	–20	36	–36	63	–63
	12	7	–7	20	–20	36	–36	63	–63

[a] Computed at ZORA-BLYP-D3(BJ)/TZ2P in $C_{\infty v}$ symmetry for X–CN and X–CC–CN and C_S symmetry for $X–C_6H_4–CN$.

Table S13. Binding and interaction energy [in kcal mol^{-1}] and intermolecular distances *R* [in Å] for several hydrogen-bonded model complexes. Further details on the definition of the intermolecular distances can be found in footnote [d].

	ZORA-DFT-D3(BJ)/TZ2P	DFT-D/TZ2P[a]			DFT-D/TZV(2d2p)[b,c]			MP2	MP2
	BLYP-D3(BJ)	BLYP-D	BP86-D	PBE-D	BLYP-D[b]	B97-D[c]	PBE-D[b]	Jurečka[d]	others
$(NH_3)_2$ (C_{2h})									
$\Delta E_{binding}$	–3.0	–3.6	–3.4	–4.0	–3.6	–3.8	–4.0		–3.0[e]
ΔE_{int}	–3.1	–3.7	–3.5	–4.0				–3.2	
R (N–N)	3.18	3.17	3.13	3.13	3.27	3.27	3.18	3.16	3.17[e]
$(H_2O)_2$ (C_s)									
$\Delta E_{binding}$	–5.2	–5.4	–5.3	–5.9	–6.0	–5.3	–6.4		–4.8[b]
ΔE_{int}	–5.2	–5.4	–5.4	–6.0				–5.0	
R (O–O)	2.90	2.90	2.86	2.87	2.93	2.93	2.89	2.91	2.92[f]
$(HCOOH)_2$ (C_{2h})									
$\Delta E_{binding}$	–17.6	–17.7	–19.2	–19.5	–16.6	–16.1	–18.5		–13.9[b]
ΔE_{int}	–21.6	–21.6	–24.8	–24.7				–18.6	
R (O–O)	2.65	2.67	2.61	2.61	2.68	2.68	2.62	2.67	2.66[f]
$(HCONH_2)_2$ (C_{2h})									
$\Delta E_{binding}$	–15.7	–15.8	–16.7	–17.0					
ΔE_{int}	–17.1	–17.3	–18.6	–18.8				–16.0	
R (N–O)	2.86	2.85	2.81	2.82				2.86	
Uracil dimer (C_{2h})									
$\Delta E_{binding}$	–19.6	–19.5	–20.5	–20.7					
ΔE_{int}	–21.8	–21.8	–23.3	–23.4				–20.7	
R (N–O)	2.78	2.78	2.74	2.74				2.80	
2-pyridoxine•2-aminopyridine (C_1)									
$\Delta E_{binding}$	–17.4	–17.5	–18.7	–18.9					
ΔE_{int}	–19.7	–20.0	–21.9	–21.9				–16.7	
R (N–O)	2.83	2.83	2.78	2.79				2.90	
R (N–N)	2.84	2.83	2.79	2.80				2.90	
Adenine•Thymine (WC) (C_S)									
$\Delta E_{binding}$	–16.7	–16.7	–17.9	–18.0	–15.5	–15.3	–16.6		–15.1[g]
ΔE_{int}	–18.5	–18.7	–20.8	–20.7					
R (N6–O4)	2.88	2.89	2.83	2.84					2.86[g]
R (N1–N3)	2.80	2.78	2.74	2.75	2.84	2.82	2.80		2.83[g]
Guanine•Cytosine (WC) (C_S)									
$\Delta E_{binding}$	–30.0	–30.1	–31.9	–31.9	–28.8	–28.5	–30.5		–27.7[g]
ΔE_{int}	–34.0	–34.2	–37.0	–36.6					
R (O6–N4)	2.75	2.74	2.70	2.70					2.75[g]
R (N1–N3)	2.89	2.89	2.84	2.86	2.93	2.91	2.89		2.90[g]
R (O2–N2)	2.88	2.88	2.83	2.85					2.89[g]

[a] T. van der Wijst, C. Fonseca Guerra, M. Swart, F. M. Bickelhaupt, B. Lippert, *Angew. Chem., Int. Ed.* **2009**, *48*, 3285. [b] S. Grimme, *J. Comput. Chem.* **2004**, *25*, 1463. [c] S. Grimme, *J. Comput. Chem.* **2006**, *27*, 1787. [d] P. Jurečka, J. Šponer, J. Cerny, P. Hobza, *Phys. Chem. Chem. Phys.* **2006**, *8*, 1985. Geometries and energies are calculated at the MP2 or CCSD(T) level of theory. See reference for the level of theory. [e] J. S. Lee, S. Y. Park, *J. Chem. Phys.* **2000**, *112*, 230. Energy calculated at the MP4 level of theory. [f] S. Tsuzuki, H. P. Lüthi, *J. Chem. Phys.* **2001**, *114*, 3949. Energy calculated at the CCSD(T) level of theory. [g] J. Šponer, P. Jurečka, P. Hobza, *J. Am. Chem. Soc.* **2004**, *126*, 10142.

Table S14. Binding and interaction energies [in kcal mol^{-1}] and intermolecular distances R [in Å] for several model van-der-Waals complexes. When the entire fragment is listed in the definition of the intermolecular distances, the geometric center of that fragment is used. Further details on the definition of the intermolecular distances can be found in footnote [d].

	ZORA-DFT-D3(BJ)/TZ2P	DFT-D/TZ2P[a]			DFT-D/TZV(2d2p)[b,c]			MP2	MP2
	BLYP-D3(BJ)	BLYP-D	BP86-D	PBE-D	BLYP-D[b]	B97-D[c]	PBE-D[b]	Jurečka[d]	others
$(CH_4)_2$ (D_{3d})									
$\Delta E_{binding}$	–0.5	–0.5	–0.2	–0.9	–0.3	–0.6	–0.6		–0.5[e]
ΔE_{int}	–0.5	–0.5	–0.2	–0.9				–0.5	
R (C–C)	3.71	3.52	3.54	3.53	3.63	3.78	3.67	3.72	3.60[e]
$(C_2H_4)_2$ (D_{2d})									
$\Delta E_{binding}$	–1.5	–1.8	–1.8	–2.2	–1.4	–1.6	–1.6		–1.3[e]
ΔE_{int}	–1.5	–1.8	–1.8	–2.2				–1.5	
R (C–C)	3.84	3.62	3.57	3.66	3.73	3.77	3.75	3.84	3.80[e]
C_6H_6•CH_4 (C_{3v})									
$\Delta E_{binding}$	–1.6	–1.6	–1.5	–1.9	–0.96	–1.5	–1.3		–1.6[b]
ΔE_{int}	–1.6	–1.6	–1.5	–1.9				–1.5	
R (Benzene–C)	3.88	3.70	3.65	3.80	3.81	3.70	3.82	3.72	3.62[b]
C_6H_6 dimer (C_{2h})									
$\Delta E_{binding}$	–3.5	–2.3	–2.4	–2.6	–2.8	–2.0	–2.0		–2.8[f]
ΔE_{int}	–3.5	–2.3	–2.4	–2.6				–2.7	
R (interplanar)	3.53	3.56	3.41	3.63	3.51	3.51	3.57	3.36	3.40[f]
C_6H_6 dimer (D_{6h})									
$\Delta E_{binding}$	–2.5	–1.4	–1.1	–0.6	–1.21	–1.77	–1.21		–1.8[f]
ΔE_{int}	–2.5	–1.4	–1.1	–0.6					
R (interplanar)	3.91	4.17	4.10	5.28	3.90	3.90	4.04		3.70[f]
pyrazine dimer (C_s)									
$\Delta E_{binding}$	–5.0	–4.2	–4.3	–4.3					
ΔE_{int}	–5.0	–4.2	–4.3	–4.4				–4.4	
R (interplanar)	3.38	3.32	3.20	3.31				3.26	
Uracil dimer (C_2)									
$\Delta E_{binding}$	–9.7	–9.5	–9.7	planar[g]					
ΔE_{int}	–10.6	–10.6	–11.0					–10.1	
R (Uracil–Uracil)	3.37	3.38	3.25					3.30	
Indole•Benzene stack (C_1)									
$\Delta E_{binding}$	–5.5								
ΔE_{int}	–5.7					–6.4		–8.1	
R (interplanar)	3.40					3.22		3.25	
Adenine•Thymine stack (C_1)									
$\Delta E_{binding}$	–11.7	–11.7	–12.3	–11.2	–11.3	–11.7	–9.4		–11.6[h]
ΔE_{int}	–12.5	–13.2	–13.7	–12.3				–12.2	
R (C4–N1)	3.47	3.39	3.26	3.40	3.44	3.36	3.49	3.31	
Guanine•Cytosine stack (C_1)									
$\Delta E_{binding}$	–12.0				–16.5	–17.5			–16.9[g]
ΔE_{int}	–13.8							–19.0	
R (C5–N1)	3.30								

[a] T. van der Wijst, C. Fonseca Guerra, M. Swart, F. M. Bickelhaupt, B. Lippert, *Angew. Chem., Int. Ed.* **2009**, *48*, 3285. [b] S. Grimme, *J. Comput. Chem.* **2004**, *25*, 1463. [c] S. Grimme, *J. Comput. Chem.* **2006**, *27*, 1787. [d] P. Jurečka, J. Šponer, J.

Cerny, P. Hobza, *Phys. Chem. Chem. Phys.* **2006**, *8*, 1985. Geometries and energies are calculated at the MP2 or CCSD(T) level of theory. See reference for the level of theory. [e] S. Tsuzuki, H. P. Lüthi, *J. Chem. Phys.* **2001**, *114*, 3949. Energy calculated at the CCSD(T) level of theory. [f] M. O. Sinnokrot, E. F. Valeev, C. D. Sherrill, *J. Am. Chem. Soc.* **2002**, *124*, 10887. Energy calculated at the CCSD(T) level of theory. [g] Geometry converged to a planar hydrogen-bonded system. [h] J. Šponer, P. Jurečka, P. Hobza, *J. Am. Chem. Soc.* **2004**, *126*, 10142. Energy calculated at the CCSD(T) level of theory.

Table S15. Binding and interaction energies [in kcal mol^{-1}] and intermolecular distances R [in Å] for other weakly bound complexes. When the entire fragment is listed in the definition of the intermolecular distances, the geometric center of that fragment is used. Further details on the definition of the intermolecular distances can be found in footnote [d].

	ZORA-DFT-D3(BJ)/TZ2P	DFT-D/TZ2P[a]			DFT-D/TZV(2d2p)[b,c]			MP2	MP2
	BLYP-D3(BJ)	BLYP-D	BP86-D	PBE-D	BLYP-D[b]	B97-D[c]	PBE-D[b]	Jurečka[d]	others
Ethylene•Acetylene (C_{2v})									
$\Delta E_{binding}$	–1.8	–1.8	–1.8	–2.3	–1.5	–1.8	–1.8		–1.5[b]
ΔE_{int}	–1.8	–1.8	–1.8	–2.3				–1.5	
R (C–C)	3.79	3.74	3.71	3.71	3.89	3.80	3.84	3.88	3.82[b]
Benzene•H_2O (C_S)									
$\Delta E_{binding}$	–3.2	–3.4	–3.5	–3.6	–3.7	–4.4	–4.2		–3.9[e]
ΔE_{int}	–3.2	–3.4	–3.6	–3.6				–3.6	
R (Benzene-O)	3.68	3.58	3.36	3.82	3.33	3.21	3.30	3.43	3.21[e]
Benzene•NH_3 (C_S)									
$\Delta E_{binding}$	–2.3	–2.4	–2.4	–2.8	–2.2	–2.9	–2.6		–2.4[b]
ΔE_{int}	–2.3	–2.5	–2.5	–2.5				–2.4	
R (Benzene–N)	3.71	3.60	3.50	3.62	3.62	3.50	3.58	3.59	3.45[b]
Benzene•HCN (C_S)									
$\Delta E_{binding}$	–4.6	–4.9	–5.1	–5.4					
ΔE_{int}	–4.6	–4.9	–5.1	–5.4				–4.5	
R (Benzene–C)	3.51	3.40	3.35	3.38				3.39	
(Benzene)$_2$ (C_{2v})									
$\Delta E_{binding}$	–3.0	–2.9	–2.9	–3.2	–2.2	–2.9	–2.3		–2.7[f
ΔE_{int}	–3.0	–2.9	–2.9	–3.2				–2.7	
R (Benzene–Benzene)	5.01	4.90	4.84	4.93	5.06	4.92	5.06	4.91	4.90[f]
Indole•Benzene, T-shaped (C_s)									
$\Delta E_{binding}$	–5.7	–6.0	–6.2	–6.3	–5.2	–6.4	–5.5		–6.2[g]
ΔE_{int}	–5.7	–6.1	–6.2	–6.3				–5.7	
R (Benzene–N)	3.36	3.29	3.24	3.28	3.34	3.34	3.34	3.24	3.16[g]
(Phenol)$_2$ (C_1)									
$\Delta E_{binding}$	–7.2	–7.3	–7.3	–7.4					
ΔE_{int}	–7.3	–7.5	–7.6	–7.7				–7.1	
R (O–O)	2.85	2.84	2.79	2.82				2.89	

[a] T. van der Wijst, C. Fonseca Guerra, M. Swart, F. M. Bickelhaupt, B. Lippert, *Angew. Chem., Int. Ed.* **2009**, *48*, 3285. [b] S. Grimme, *J. Comput. Chem.* **2004**, *25*, 1463. [c] S. Grimme, *J. Comput. Chem.* **2006**, *27*, 1787. [d] P. Jurečka, J. Šponer, J. Cerny, P. Hobza, *Phys. Chem. Chem. Phys.* **2006**, *8*, 1985. Geometries and energies are calculated at the MP2 or CCSD(T) level of theory. See reference for the level of theory. [e] D. Feller, *J. Phys. Chem. A* **1999**, *103*, 7558. [f] M. O. Sinnokrot, E. F. Valeev, C. D. Sherrill, *J. Am. Chem. Soc.* **2002**, *124*, 10887. Energy calculated at the CCSD(T) level of theory. [g] J. Braun, H. J. Neusser, P. Hobza, *J. Phys. Chem. A* **2003**, *107*, 3918.

Table S16. Cartesian coordinates [in Å] and applied point group symmetry of all the infinite X–CN, X–CC–CN, and X–C_6H_4–CN systems, computed at ZORA-BLYP-D3(BJ)/TZ2P.

Cl-CN ($C_{\infty v}$)				Br–CN ($C_{\infty v}$)			
C	2.99192493	0.00000000	0.00000000	C	3.06644348	0.00000000	0.00000000
N	4.15726503	0.00000000	0.00000000	N	4.23105030	0.00000000	0.00000000
Cl	1.34706578	0.00000000	0.00000000	Br	1.24798526	0.00000000	0.00000000
VEC1[a]	5.67807036	0.00000000	0.00000000	VEC1	5.73239860	0.00000000	0.00000000
I-CN ($C_{\infty v}$)				H-CN ($C_{\infty v}$)			
C	3.30293321	0.00000000	0.00000000	C	2.09913229	0.00000000	0.00000000
N	4.46907139	0.00000000	0.00000000	N	3.25455560	0.00000000	0.00000000
I	1.25238090	0.00000000	0.00000000	H	1.00975659	0.00000000	0.00000000
VEC1	6.03102088	0.00000000	0.00000000	VEC1	4.25270691	0.00000000	0.00000000
Cl-CC-CN ($C_{\infty v}$)				I-CC-CN ($C_{\infty v}$)			
C	-0.03040429	0.00000000	0.00000000	C	-0.04150663	0.00000000	0.00000000
C	1.18761026	0.00000000	0.00000000	C	1.18310091	0.00000000	0.00000000
C	2.54730894	0.00000000	0.00000000	C	2.54182912	0.00000000	0.00000000
N	3.71759241	0.00000000	0.00000000	N	3.71323001	0.00000000	0.00000000
Cl	-1.66114650	0.00000000	0.00000000	I	-2.05685893	0.00000000	0.00000000
VEC1	8.29033849	0.00000000	0.00000000	VEC1	8.64508849	0.00000000	0.00000000
Br-CC-CN ($C_{\infty v}$)				H-CC-CN ($C_{\infty v}$)			
C	-0.08420105	0.00000000	0.00000000	C	-1.20835697	0.00000000	0.00000000
C	1.14437776	0.00000000	0.00000000	C	0.00381303	0.00000000	0.00000000
C	2.51442804	0.00000000	0.00000000	C	1.36731060	0.00000000	0.00000000
N	3.69269970	0.00000000	0.00000000	N	2.53506413	0.00000000	0.00000000
Br	-1.89752588	0.00000000	0.00000000	H	-2.28632752	0.00000000	0.00000000
VEC1	8.40577593	0.00000000	0.00000000	VEC1	7.00915987	0.00000000	0.00000000
Cl-C_6H_4-CN (C_s)				Br-C_6H_4-CN (C_s)			
C	4.53597382	-0.00272002	0.00000000	C	4.60476449	-0.00355373	0.00000000
C	3.82754183	-1.22088288	0.00000000	C	3.89596361	-1.22179687	0.00000000
C	2.43535065	-1.22040215	0.00000000	C	2.50321989	-1.21962233	0.00000000
C	1.74369994	-0.00260411	0.00000000	C	1.81451685	-0.00138394	0.00000000
C	2.43544962	1.21514025	0.00000000	C	2.50497859	1.21586014	0.00000000
C	3.82763772	1.21550658	0.00000000	C	3.89782001	1.21575233	0.00000000
C	5.96463767	-0.00266245	0.00000000	C	6.03333198	-0.00436108	0.00000000
H	1.88304328	-2.15518980	0.00000000	H	1.95356291	-2.15551242	0.00000000
H	1.88321559	2.14997235	0.00000000	H	1.95704854	2.15279312	0.00000000
H	4.37346058	2.15463097	0.00000000	H	4.44369151	2.15497771	0.00000000
H	4.37329239	-2.16004522	0.00000000	H	4.44024993	-2.16192118	0.00000000
Cl	-0.00778444	-0.00254069	0.00000000	Br	-0.10509648	-0.00091025	0.00000000
N	7.12820200	-0.00245147	0.00000000	N	7.19677979	-0.00457014	0.00000000
VEC1	10.2488005	0.00000000	0.00000000	VEC1	10.4189328	0.00000000	0.00000000
I-C_6H_4-CN (C_s)				H-C_6H_4-CN (C_s)			
C	4.71276447	-0.00298779	0.00000000	C	4.41462769	-0.00434715	0.00000000
C	4.00281192	-1.22069868	0.00000000	C	3.70693422	-1.22309941	0.00000000
C	2.60896903	-1.21810341	0.00000000	C	2.31230599	-1.21320419	0.00000000
C	1.91034706	-0.00212827	0.00000000	C	1.61344434	0.00004076	0.00000000
C	2.60969776	1.21342501	0.00000000	C	2.31605691	1.21106253	0.00000000
C	4.00354886	1.21515471	0.00000000	C	3.71070933	1.21659366	0.00000000
C	6.14193159	-0.00337316	0.00000000	C	5.84635194	-0.00642477	0.00000000
H	2.06841955	-2.15993187	0.00000000	H	1.76764346	-2.15416102	0.00000000
H	2.06974839	2.15559773	0.00000000	H	1.77432481	2.15367963	0.00000000
H	4.54846727	2.15526635	0.00000000	H	4.26199001	2.15251881	0.00000000
H	4.54713277	-2.16115470	0.00000000	H	4.25532610	-2.16072675	0.00000000
I	-0.22404592	-0.00173230	0.00000000	H	0.52678188	0.00155288	0.00000000
N	7.30564736	-0.00358226	0.00000000	N	7.01043030	-0.00773362	0.00000000
VEC1	10.6877355	0.00000000	0.00000000	VEC1	10.0441201	0.00000000	0.00000000

[a] Vec1: Coordinates of the lattice vector.

Table S17. Cartesian coordinates [in Å], applied point group symmetry and total ADF bonding energies [in kcal mol^{-1}] of all optimized molecular fragments included in the extended S22 benchmark set, computed at ZORA-BLYP-D3(BJ)/TZ2P.

NH_3 (C_{3v})			[-434.78]
N	0.00000000	0.00000000	-0.56153528
H	0.47306494	-0.81937251	-0.17540291
H	0.47306494	0.81937251	-0.17540291
H	-0.94612988	0.00000000	-0.17540291

H_2O (C_{2v})			[-318.00]
O	0.00000000	0.00000000	0.36656121
H	0.00000000	0.76837678	-0.22933908
H	0.00000000	-0.76837678	-0.22933908

HCOOH (C_s)			[-664.77]
C	-0.29586279	-0.32590570	0.00000000
O	0.95838022	0.21485954	0.00000000
O	-1.32905222	0.30047539	0.00000000
H	-0.21327318	-1.42557392	0.00000000
H	0.84875923	1.19042228	0.00000000

$HCONH_2$ (C_s)			[-783.09]
C	-0.60546236	0.27953031	0.00000000
O	-1.66249027	-0.33379574	0.00000000
N	0.63574140	-0.29657419	0.00000000
H	1.47558616	0.26781309	0.00000000
H	0.71775154	-1.30742373	0.00000000
H	-0.55063208	1.38958221	0.00000000

2-pyridoxine (C_s)			[-1731.73]
O	2.68041559	-0.00270928	0.00000000
N	0.67905495	-1.14476084	0.00000000
C	-0.74820325	1.18566195	0.00000000
C	-1.43427267	-0.06645102	0.00000000
C	-0.68666121	-1.21259314	0.00000000
C	1.44705803	0.05635408	0.00000000
C	0.62120859	1.25226286	0.00000000
H	-1.32941186	2.10579661	0.00000000
H	-2.51675848	-0.12041939	0.00000000
H	-1.11891595	-2.20786293	0.00000000
H	1.15093702	2.19985330	0.00000000
H	1.23480181	-1.99593319	0.00000000

Pyrazine (D_{2h})			[-1464.35]
C	-0.69972229	-1.13769200	0.00000000
C	0.69972229	-1.13769200	0.00000000
N	1.41466316	0.00000000	0.00000000
C	0.69972229	1.13769200	0.00000000
C	-0.69972229	1.13769200	0.00000000
N	-1.41466316	0.00000000	0.00000000
H	-1.25764496	-2.07329096	0.00000000
H	1.25764496	-2.07329096	0.00000000
H	1.25764496	2.07329096	0.00000000
H	-1.25764496	2.07329096	0.00000000

Adenine (C_1)			[-2306.55]
N	-1.97914747	-0.55614436	0.00716522
C	-1.21565599	0.55819761	0.00003934
C	0.18999980	0.41983093	-0.00350905
C	0.68228819	-0.89755935	-0.00426642
N	-0.04655809	-2.02526317	-0.00266242
C	-1.36456202	-1.75670044	0.00342175
N	1.23111708	1.34756342	0.00306274
C	2.32442765	0.60961433	0.00445522
N	2.06063269	-0.75401458	-0.00095788
N	-1.82686263	1.77560057	-0.02610745
H	-2.02593722	-2.62003569	0.00811653
H	3.33724465	0.98912968	0.00937913
H	2.73306678	-1.51089860	0.00138796
H	-2.82570361	1.81068834	0.12854068
H	-1.27155531	2.60705914	0.12183585

HCN ($C_{\infty v}$)			[-439.95]
H	0.00000000	0.00000000	-0.52034358
C	0.00000000	0.00000000	0.55134095
N	0.00000000	0.00000000	1.70756349

CH_4 (T_d)			[-536.29]
C	0.00000000	0.00000000	0.00000000
H	0.63195459	0.63195459	-0.63195459
H	0.63195459	-0.63195459	0.63195459
H	-0.63195459	0.63195459	0.63195459
H	-0.63195459	-0.63195459	-0.63195459

C_2H_2 ($D_{\infty h}$)			[-510.55]
C	0.00000000	0.00000000	-0.60233807
C	0.00000000	0.00000000	0.60233807
H	0.00000000	0.00000000	-1.66921133
H	0.00000000	0.00000000	1.66921133

C_2H_4 (D_{2h})			[-711.47]
C	0.66690922	0.00000000	0.00000000
C	-0.66690922	0.00000000	0.00000000
H	1.23927635	0.92597205	0.00000000
H	1.23927635	-0.92597205	0.00000000
H	-1.23927635	0.92597205	0.00000000
H	-1.23927635	-0.92597205	0.00000000

C_6H_6 (D_{6h})			[-1681.10]
C	0.00000000	-1.39991182	0.00000000
C	1.21235920	-0.69995591	0.00000000
C	1.21235920	0.69995591	0.00000000
C	0.00000000	1.39991182	0.00000000
C	-1.21235920	0.69995591	0.00000000
C	-1.21235920	-0.69995591	0.00000000
H	2.15429475	-1.24378265	0.00000000
H	2.15429475	1.24378265	0.00000000
H	0.00000000	2.48756530	0.00000000
H	-2.15429475	1.24378265	0.00000000
H	-2.15429475	-1.24378265	0.00000000
H	0.00000000	-2.48756530	0.00000000

2-aminopyridine (C_1)			[-1847.14]
N	0.32886752	1.33176947	-0.00507591
C	0.93033794	0.12656094	-0.00163468
C	0.20546778	-1.08703124	0.00227617
C	-1.18248284	-1.02988203	-0.00211550
C	-1.82045509	0.21934902	-0.00208533
C	-1.01579577	1.35893036	-0.00013362
H	0.72932050	-2.04066327	0.01388023
H	-1.76424002	-1.94861579	-0.00321643
H	-2.90314247	0.30468203	-0.00486516
H	-1.46585660	2.35089340	-0.00021116
N	2.32096164	0.13448686	0.04634958
H	2.79860019	-0.67475613	-0.33114546
H	2.73533332	1.02627557	-0.20394226

Uracil (C_s)			[-1797.65]
O	-2.28355644	-1.37691904	0.00000000
C	-1.23438694	-0.74882234	0.00000000
N	-1.19072996	0.65403201	0.00000000
C	-0.02108583	1.38394010	0.00000000
C	1.19405678	0.78672550	0.00000000
C	1.29563938	-0.67023815	0.00000000
N	0.03081509	-1.32372527	0.00000000
O	2.33057220	-1.32839187	0.00000000
H	2.11158548	1.36108922	0.00000000
H	-2.09527010	1.11060278	0.00000000
H	0.05203307	-2.34103527	0.00000000
H	-0.14630735	2.46183937	0.00000000

Thymine (C_s)			[-2167.31]
N	1.58287880	-0.87632164	0.00000000
C	0.22908351	-1.16386365	0.00000000
C	-0.72425202	-0.19969068	0.00000000
C	-0.29554310	1.20617595	0.00000000
N	1.11022975	1.39077205	0.00000000
C	2.10528726	0.41936131	0.00000000
C	-2.20185473	-0.48568124	0.00000000
O	-1.04678408	2.17823431	0.00000000
O	3.30631019	0.65727944	0.00000000
H	2.27302588	-1.61769561	0.00000000
H	1.43467969	2.35490306	0.00000000
H	-0.01530252	-2.22189888	0.00000000
H	-2.68137522	-0.03918930	0.87908169
H	-2.68137522	-0.03918930	-0.87908169
H	-2.39503324	-1.56333556	0.00000000

Guanine (C_1)			[-2461.92]
O	-0.02828219	2.96311466	0.00000000
C	0.00963844	1.73959828	0.00000000
N	1.30493041	1.07510996	0.00000000
C	1.52926385	-0.28207513	0.00000000
N	0.55791316	-1.17610256	0.00000000
C	-0.67788881	-0.61061288	0.00000000
C	-1.03338880	0.74800606	0.00000000
N	-2.41657760	0.89579362	0.00000000
C	-2.88758923	-0.33122707	0.00000000
N	-1.87584257	-1.29091629	0.00000000
N	2.82913353	-0.71157966	0.00000000
H	2.08536732	1.72648663	0.00000000
H	-3.93216381	-0.61010480	0.00000000
H	-1.97709962	-2.29833749	0.00000000
H	2.99547837	-1.70729694	0.00000000
H	3.61170314	-0.07719673	0.00000000

Cytosine (C_1)			[-1904.30]
C	1.58578279	-0.74282203	0.00000000
N	1.60924096	0.69966787	0.00000000
C	0.48785054	1.46570954	0.00000000
C	-0.73902881	0.87216870	0.00000000
C	-0.75189565	-0.56970335	0.00000000
N	0.33932251	-1.32344027	0.00000000
O	2.65782082	-1.33823431	0.00000000
N	-1.95104053	-1.22251333	0.00000000
H	2.53458096	1.11573385	0.00000000
H	0.62491599	2.54235694	0.00000000
H	-1.65010239	1.45998463	0.00000000
H	-2.82856440	-0.72623851	0.00000000
H	-1.94440988	-2.23393453	0.00000000

Indole (C_s)			[-2337.16]
C	0.30542070	-0.74351954	0.00000000
C	1.68410278	-1.16179565	0.00000000
H	2.05501625	-2.17766325	0.00000000
C	2.45706930	-0.02430811	0.00000000
N	1.63026064	1.09397508	0.00000000
C	0.30372896	0.68630226	0.00000000
C	-0.87962200	1.43424284	0.00000000
H	-0.86147816	2.52195138	0.00000000
C	-2.08512985	0.73358147	0.00000000
H	-3.02202114	1.28414466	0.00000000
H	1.94737677	2.05224389	0.00000000
H	3.53163628	0.08979364	0.00000000
H	-3.06716201	-1.19340529	0.00000000
C	-2.10925027	-0.67994777	0.00000000
C	-0.92997157	-1.42074477	0.00000000
H	-0.95960782	-2.50796066	0.00000000

Phenol (C_s)			[-1828.07]
C	-1.12974832	0.06047284	0.00000000
O	-2.51459521	0.12065923	0.00000000
H	-2.86667234	-0.78573658	0.00000000
C	-0.43040254	-1.15390883	0.00000000
C	0.96857652	-1.14721133	0.00000000
C	1.67159768	0.06218002	0.00000000
C	0.96135435	1.26960191	0.00000000
C	-0.43513509	1.27622513	0.00000000
H	-0.97728164	-2.09645726	0.00000000
H	1.50622138	-2.09212363	0.00000000
H	2.75806646	0.06451316	0.00000000
H	1.49731678	2.21547151	0.00000000
H	-0.99795065	2.20562629	0.00000000

Table S18. Cartesian coordinates [in Å], applied point group symmetry and total ADF bonding energies [in kcal mol^{-1}] of all optimized molecular complexes included in the extended S22 benchmark set, computed at ZORA-BLYP-D3(BJ)/TZ2P.

$(NH_3)_2$ (C_{2h})			[-872.60]
N	-1.58869617	-0.04555697	0.00000000
H	-2.17315641	0.12958008	-0.81934602
H	-2.17315641	0.12958008	0.81934602
H	-0.85076755	0.66371143	0.00000000
N	1.58869617	0.04555697	0.00000000
H	2.17315641	-0.12958008	-0.81934602
H	0.85076755	-0.66371143	0.00000000
H	2.17315641	-0.12958008	0.81934602

$(H_2O)_2$ (C_s)			[-641.19]
O	-1.55408083	-0.11590217	0.00000000
H	-1.95349719	0.76963466	0.00000000
H	-0.58700091	0.04833385	0.00000000
O	1.35609525	0.11429812	0.00000000
H	1.66466258	-0.39259986	-0.77118104
H	1.66466258	-0.39259986	0.77118104

$(HCOOH)_2$ (C_{2h})			[-1347.17]
C	-1.88542600	-0.18448300	0.00000000
O	-1.46914500	1.07118000	0.00000000
O	-1.18283200	-1.19649600	0.00000000
H	-2.98474000	-0.24691300	0.00000000
H	-0.44832400	1.11219600	0.00000000
C	1.88542600	0.18448300	0.00000000
O	1.46914500	-1.07118000	0.00000000
O	1.18283200	1.19649600	0.00000000
H	2.98474000	0.24691300	0.00000000
H	0.44832400	-1.11219600	0.00000000

$(HCONH_2)_2$ (C_{2h})			[-1581.89]
C	-2.01923437	0.05308640	0.00000000
O	-1.45445643	1.15621955	0.00000000
N	-1.39876273	-1.14350231	0.00000000
H	-1.95247802	-1.99062305	0.00000000
H	-0.36384094	-1.20791161	0.00000000
H	-3.12495635	-0.01777189	0.00000000
C	2.01923437	-0.05308640	0.00000000
O	1.45445643	-1.15621955	0.00000000
N	1.39876273	1.14350231	0.00000000
H	1.95247802	1.99062305	0.00000000
H	0.36384094	1.20791161	0.00000000
H	3.12495635	0.01777189	0.00000000

Uracil dimer (C_{2h})			[-3614.92]
O	-1.49091225	0.99370184	0.00000000
C	-0.64582186	1.90634821	0.00000000
N	0.71547379	1.67253423	0.00000000
C	1.64160461	2.69119602	0.00000000
C	1.28513106	3.99945131	0.00000000
C	-0.12302885	4.37122986	0.00000000
N	-0.99680493	3.23995760	0.00000000
O	-0.59033963	5.50450928	0.00000000
H	2.02217408	4.79257544	0.00000000
H	1.02118197	0.67784606	0.00000000
H	-1.99394129	3.44278401	0.00000000
H	2.67616590	2.36373431	0.00000000
O	1.49091225	-0.99370184	0.00000000
C	0.64582186	-1.90634821	0.00000000
N	-0.71547379	-1.67253423	0.00000000
C	-1.64160461	-2.69119602	0.00000000
C	-1.28513106	-3.99945131	0.00000000
C	0.12302885	-4.37122986	0.00000000
N	0.99680493	-3.23995760	0.00000000
O	0.59033963	-5.50450928	0.00000000
H	-2.02217408	-4.79257544	0.00000000
H	-1.02118197	-0.67784606	0.00000000
H	1.99394129	-3.44278401	0.00000000
H	-2.67616590	-2.36373431	0.00000000

Uracil stacked dimer (C_2)			[-3604.99]
N	2.03260029	-1.19966064	-0.14138307
C	2.08940899	-0.63514849	-1.39480817
H	2.37222748	-1.31071547	-2.19504801
C	1.80256678	0.67255571	-1.60296870
H	1.84473506	1.11437132	-2.59021959
C	1.36501431	1.51014830	-0.49259295
O	0.97151096	2.67332797	-0.57151234
N	1.39923247	0.84903989	0.76413228
H	1.02824078	1.36958613	1.55859803
C	1.63987146	-0.49935423	1.00532110
O	1.51068503	-1.03983846	2.09644219
H	2.11390876	-2.20321832	-0.01982198
N	-2.03260029	1.19966064	-0.14138307
C	-2.08940899	0.63514849	-1.39480817
H	-2.37222748	1.31071547	-2.19504801
C	-1.80256678	-0.67255571	-1.60296870
H	-1.84473506	-1.11437132	-2.59021959
C	-1.36501431	-1.51014830	-0.49259295
O	-0.97151096	-2.67332797	-0.57151234
N	-1.39923247	-0.84903989	0.76413228
H	-1.02824078	-1.36958613	1.55859803
C	-1.63987146	0.49935423	1.00532110
O	-1.51068503	1.03983846	2.09644219
H	-2.11390876	2.20321832	-0.01982198

Indole Benzene stack (C_1)			[-4023.81]
C	0.00538540	1.50460871	-1.47962481
C	-1.24367930	0.92277461	-1.71972080
C	-1.33295359	-0.26388371	-2.45497044
C	-0.17254271	-0.86956471	-2.95167652
C	1.07761453	-0.28379523	-2.71517643
C	1.16562280	0.90394907	-1.98004142
H	0.07606003	2.41650488	-0.89264443
H	-2.14247010	1.38279097	-1.31718707
H	-2.30390906	-0.71994195	-2.63454752
H	-0.24218283	-1.79129545	-3.52572670
H	1.97978686	-0.75004731	-3.10678542
H	2.13589891	1.35456352	-1.78606144
H	-2.94744825	0.86856673	2.50739642
C	-2.03947695	0.41195728	2.12155437
C	-0.81279445	1.04086727	2.32246206
H	-0.75664693	1.98415042	2.86138740
C	0.35654222	0.43814757	1.81856185
C	1.75340071	0.79095219	1.81575481
H	2.20454687	1.66521266	2.26531443
C	2.42362163	-0.20188395	1.13845401
N	1.52009229	-1.17713602	0.73642955
C	0.24071260	-0.80395704	1.11973361
C	-0.98834548	-1.44191869	0.92319412
H	-1.05531365	-2.38307291	0.38283102
C	-2.12646838	-0.81827590	1.42997201
H	-3.09806717	-1.28492515	1.29028526
H	1.72966733	-1.92814160	0.09495570
H	3.47572125	-0.30555180	0.91476737

2-pyridoxine•2-aminopyridine (C_1) [-3596.28]

O	-1.42592162	-1.95947675	-0.06509749
N	-1.42869057	0.34469615	0.01958723
C	-4.16979980	0.41067198	-0.02458932
C	-3.43493228	1.62755849	0.03403152
C	-2.06513069	1.54626334	0.05426094
C	-2.08601966	-0.89513695	-0.03740551
C	-3.52904617	-0.80430078	-0.05907440
H	-5.25740495	0.44567710	-0.04202642
H	-3.93000262	2.59161085	0.06208857
H	-1.41960432	2.41826279	0.09846984
H	-4.07440571	-1.74201536	-0.10340807
H	-0.37465946	0.32660784	0.03336942
N	1.40881857	0.35983331	0.03756595
C	2.11042344	-0.80435482	0.04359370
C	3.53189281	-0.79810526	-0.00148309
C	4.20247407	0.41115190	-0.04959431
C	3.47355810	1.61511944	-0.05367182
C	2.08812672	1.52254616	-0.00969549
H	4.07321502	-1.74167533	0.00385048
H	5.28932099	0.42643738	-0.08438566
H	3.96610053	2.58148776	-0.09083301
H	1.47570790	2.42365064	-0.01344674
N	1.40020086	-1.96241858	0.11125152
H	1.88755000	-2.84127895	0.02047537
H	0.36836295	-1.96110579	0.04347304

Adenine•Thymine (WC) (C_s) [-4490.55]

N	-1.25247403	0.60540189	0.00000000
C	-1.99687470	-0.53264652	0.00000000
C	-3.40469661	-0.38445648	0.00000000
C	-3.89901741	0.93049003	0.00000000
N	-3.17798389	2.06749459	0.00000000
C	-1.86283128	1.81126049	0.00000000
N	-4.44220797	-1.31397855	0.00000000
C	-5.53823925	-0.57983578	0.00000000
N	-5.27606509	0.78433688	0.00000000
N	-1.37606510	-1.72841392	0.00000000
H	-1.18882212	2.66479614	0.00000000
H	-6.55003721	-0.96181338	0.00000000
H	-5.94852195	1.54124913	0.00000000
H	-0.34931694	-1.78484206	0.00000000
H	-1.94011155	-2.56652142	0.00000000
N	3.55545101	1.73988518	0.00000000
C	4.26007685	0.55309422	0.00000000
C	3.64301327	-0.65587276	0.00000000
C	2.17702331	-0.67583497	0.00000000
N	1.54621424	0.56932277	0.00000000
C	2.15638160	1.81090515	0.00000000
C	4.37853763	-1.96924862	0.00000000
O	1.50714844	-1.72454992	0.00000000
O	1.55344315	2.88049415	0.00000000
H	4.03342927	2.63311677	0.00000000
H	0.48500405	0.57871412	0.00000000
H	5.34127177	0.65500880	0.00000000
H	4.10645360	-2.56541398	0.87925719
H	4.10645360	-2.56541398	-0.87925719
H	5.46251131	-1.81405415	0.00000000

Adenine Thymine stack (C_1) [-4485.58]

N	0.38399084	2.52439440	-0.55724358
C	-1.00289483	2.54688808	-0.58535304
H	-1.54095892	3.12691511	-1.32205008
N	-1.55089242	1.79410168	0.35002016
C	-0.46516627	1.23805186	1.02711175
C	-0.37120957	0.31795823	2.09520487
N	-1.47231573	-0.20481365	2.70765414
H	-1.30664098	-1.01263111	3.29658703
H	-2.33061514	-0.21339492	2.16754212
N	0.85740253	-0.04556484	2.53241933
C	1.93818351	0.46238575	1.90942150
H	2.89976662	0.13369616	2.29796820
N	1.98661498	1.31335056	0.86653267
C	0.75364389	1.67567017	0.47299787
H	1.00988552	2.96792961	-1.21774969
N	1.22209949	-0.67814006	-2.02954320
C	1.40469796	-1.60452272	-1.01936131
H	2.43599568	-1.89192840	-0.84210205
C	0.37504359	-2.11035613	-0.29613165
C	0.55903201	-3.10044099	0.82140700
H	0.34925658	-2.62865593	1.78869283
H	-0.13041667	-3.94387165	0.70709095
H	1.58651077	-3.47798019	0.84334229
C	-0.98031479	-1.63303173	-0.59013282
O	-2.00159335	-1.96349599	0.01336764
N	-1.06116406	-0.71696224	-1.66814971
H	-1.98105585	-0.31805922	-1.83839949
C	-0.01723600	-0.15171363	-2.38549818
O	-0.15505603	0.71917162	-3.24164913
H	2.01779918	-0.27383623	-2.50794677

Guanine Cytosine Stack (C_1) [-4378.17]

N	-2.10796955	1.81527127	0.98143990
N	0.12948599	1.12124095	1.19158291
C	-1.20731594	0.81102181	1.29826248
N	-1.64622214	-0.36361589	1.69935952
C	-0.64570682	-1.25367341	1.91311576
C	0.74178384	-1.07501177	1.77631501
C	1.23854481	0.21428656	1.37251230
N	-0.80777506	-2.58726831	2.21292462
C	0.46061111	-3.15927361	2.21624237
N	1.41152330	-2.28104868	1.96306768
O	2.38143297	0.60585130	1.14850296
H	-1.85002551	2.35671076	0.15882411
H	-3.05007946	1.43997090	0.90354865
H	0.40548613	2.05575704	0.90270139
H	-1.70197101	-3.06248975	2.25451422
H	0.60910816	-4.20933174	2.42665307
C	0.77467171	-0.08319985	-1.90301809
N	-1.61645623	-0.55377704	-1.76955944
C	-1.36536353	-1.84076950	-1.33478288
N	0.01700082	-2.20915990	-1.24134732
C	1.04172440	-1.35443166	-1.48470749
C	-0.60953204	0.26581075	-2.02341884
N	-0.94802190	1.57320138	-2.36419158
O	-2.21755597	-2.67007001	-1.02905706
H	1.57323063	0.63132985	-2.06485864
H	0.19697467	-3.13456248	-0.86462702
H	2.04540412	-1.72270605	-1.30347409
H	-0.26332438	2.08685168	-2.90889450
H	-1.90226380	1.66164538	-2.70327380

Ethylene•Acetylene (C_{2v}) [-1223.81]

C	0.00000000	-0.66701651	-2.08164745
C	0.00000000	0.66701651	-2.08164745
H	0.92578334	-1.23932934	-2.08084350
H	-0.92578334	-1.23932934	-2.08084350
H	-0.92578334	1.23932934	-2.08084350
H	0.92578334	1.23932934	-2.08084350
C	0.00000000	0.00000000	2.85298891
C	0.00000000	0.00000000	1.64740361
H	0.00000000	0.00000000	0.57750415
H	0.00000000	0.00000000	3.91965667

Guanine•Cytosine (WC) (C_s) [-4396.18]

C	2.71600759	-1.07047818	0.00000000
N	4.12434167	-0.89220200	0.00000000
C	4.70793852	0.34169195	0.00000000
C	3.93672283	1.46102452	0.00000000
C	2.50357927	1.27913267	0.00000000
N	1.94867487	0.05293879	0.00000000
O	2.27032753	-2.23033736	0.00000000
N	1.67810476	2.33574291	0.00000000
H	4.67791362	-1.74199439	0.00000000
H	5.79226085	0.36744060	0.00000000
H	4.38163892	2.44963245	0.00000000
H	2.06245531	3.26989332	0.00000000
H	0.63809715	2.20659649	0.00000000
O	-1.04903129	2.02132477	0.00000000
C	-1.65448082	0.92843932	0.00000000
N	-0.92195491	-0.28323206	0.00000000
C	-1.46162345	-1.55208963	0.00000000
N	-2.77298011	-1.78685265	0.00000000
C	-3.50380520	-0.65107594	0.00000000
C	-3.06393369	0.68615732	0.00000000
N	-4.14603601	1.56452966	0.00000000
C	-5.20711351	0.78991563	0.00000000
N	-4.88076609	-0.56763518	0.00000000
N	-0.58830727	-2.58766504	0.00000000
H	0.11417251	-0.18226555	0.00000000
H	-6.23578208	1.12339776	0.00000000
H	-5.51343266	-1.35779904	0.00000000
H	-0.98292549	-3.51670962	0.00000000
H	0.43080717	-2.45796345	0.00000000

$(CH_4)_2$ (D_{3d}) [-1073.09]

C	0.00000000	0.00000000	1.85459551
H	-0.89281947	0.51546956	1.48766524
H	0.89281947	0.51546956	1.48766524
H	0.00000000	-1.03093912	1.48766524
H	0.00000000	0.00000000	2.94904591
C	0.00000000	0.00000000	-1.85459551
H	0.00000000	0.00000000	-2.94904591
H	-0.89281947	-0.51546956	-1.48766524
H	0.89281947	-0.51546956	-1.48766524
H	0.00000000	1.03093912	-1.48766524

$(C_2H_4)_2$ (D_{2d}) [-1424.46]

C	-0.47161041	-0.47161041	-1.86254864
C	0.47161041	0.47161041	-1.86254864
H	-0.87680683	-0.87680683	-0.93747642
H	0.87680683	0.87680683	-0.93747642
H	-0.87569719	-0.87569719	-2.78903462
H	0.87569719	0.87569719	-2.78903462
C	-0.47161041	0.47161041	1.86254864
C	0.47161041	-0.47161041	1.86254864
H	-0.87680683	0.87680683	0.93747642
H	0.87680683	-0.87680683	0.93747642
H	-0.87569719	0.87569719	2.78903462
H	0.87569719	-0.87569719	2.78903462

C_6H_6•CH_4 (C_{3v}) [-2218.96]

C	0.69978678	1.21206626	-1.12254416
C	1.39955771	0.00000000	-1.12254281
C	0.69978678	-1.21206626	-1.12254416
C	-0.69977886	-1.21205253	-1.12254281
C	-1.39957356	0.00000000	-1.12254416
C	-0.69977886	1.21205253	-1.12254281
H	1.24349804	2.15380178	-1.12022488
H	2.48697798	0.00000000	-1.12019402
H	1.24349804	-2.15380178	-1.12022488
H	-1.24348899	-2.15378611	-1.12019402
H	-2.48699608	0.00000000	-1.12022488
H	-1.24348899	2.15378611	-1.12019402
C	0.00000000	0.00000000	2.76036039
H	-0.51581208	0.89341272	3.12747438
H	-0.51581208	-0.89341272	3.12747438
H	0.00000000	0.00000000	1.66651588
H	1.03162415	0.00000000	3.12747438

Benzene•H_2O (C_s) [-2002.32]

C	0.95594564	-0.62847781	-1.21274726
C	0.40392072	0.65787045	-1.21332053
C	0.12875741	1.30212300	0.00000000
C	0.40392072	0.65787045	1.21332053
C	0.95594564	-0.62847781	1.21274726
C	1.23270587	-1.27079559	0.00000000
H	1.17011031	-1.12824662	-2.15438507
H	0.18575339	1.15649270	-2.15431453
H	-0.29837025	2.30197561	0.00000000
H	0.18575339	1.15649270	2.15431453
H	1.17011031	-1.12824662	2.15438507
H	1.66363794	-2.26930902	0.00000000
O	-3.00081261	-0.02240330	0.00000000
H	-3.02859840	-0.99409956	0.00000000
H	-2.04670068	0.18417012	0.00000000

Benzene•NH_3 (C_s) [-2118.22]

C	-0.72293129	0.50557489	-1.21238420
C	-1.40681476	0.35509738	0.00000000
C	-0.72293129	0.50557489	1.21238420
C	0.64425644	0.80671040	1.21218929
C	1.32773162	0.95750077	0.00000000
C	0.64425644	0.80671040	-1.21218929
H	-1.25392361	0.38693110	-2.15377379
H	-2.46751354	0.11705207	0.00000000
H	-1.25392361	0.38693110	2.15377379
H	1.17513515	0.92403807	2.15398721
H	2.38961916	1.19173815	0.00000000
H	1.17513515	0.92403807	-2.15398721
N	0.12369798	-3.04944578	0.00000000
H	0.71589393	-3.21024908	-0.81752053
H	0.71589393	-3.21024908	0.81752053
H	-0.09719463	-2.05027438	0.00000000

Benzene•HCN (C_s) [-2125.68]

C	-0.71408359	-1.02242634	1.21268132
C	-1.41375617	-0.99997970	0.00000000
C	-0.71408359	-1.02242634	-1.21268132
C	0.68554083	-1.06723791	-1.21283278
C	1.38547183	-1.08958345	0.00000000
C	0.68554083	-1.06723791	1.21283278
H	-1.25727913	-1.00340551	2.15408654
H	-2.50014843	-0.96311929	0.00000000
H	-1.25727913	-1.00340551	-2.15408654
H	1.22884718	-1.08391752	-2.15424790
H	2.47194683	-1.12412482	0.00000000
H	1.22884718	-1.08391752	2.15424790
N	0.00292141	3.61575510	0.00000000
C	0.07678056	2.46132148	0.00000000
H	0.14677021	1.38855443	0.00000000

Benzene dimer (C_{2v}, T-shaped) [-3365.16]

C	0.00000000	0.00000000	1.10518336
C	0.00000000	-1.21117326	1.80614978
C	0.00000000	-1.21251510	3.20631894
C	0.00000000	0.00000000	3.90687536
C	0.00000000	1.21251510	3.20631894
C	0.00000000	1.21117326	1.80614978
H	0.00000000	0.00000000	0.02012071
H	0.00000000	-2.15183823	1.25960587
H	0.00000000	-2.15459899	3.75044334
H	0.00000000	0.00000000	4.99460836
H	0.00000000	2.15459899	3.75044334
H	0.00000000	2.15183823	1.25960587
C	-1.39971703	0.00000000	-2.50632730
C	-0.69979819	1.21196027	-2.50663932
C	0.69979819	1.21196027	-2.50663932
C	1.39971703	0.00000000	-2.50632730
C	0.69979819	-1.21196027	-2.50663932
C	-0.69979819	-1.21196027	-2.50663932
H	-2.48681319	0.00000000	-2.49978302
H	-1.24381311	2.15338888	-2.50221809
H	1.24381311	2.15338888	-2.50221809
H	2.48681319	0.00000000	-2.49978302
H	1.24381311	-2.15338888	-2.50221809
H	-1.24381311	-2.15338888	-2.50221809

$(C_6H_6)_2$ (C_{2h}) [-3365.68]

C	-1.13568505	-1.44656968	0.00000000
C	-1.54059038	-0.87585546	1.21105772
C	-1.54059038	-0.87585546	-1.21105772
C	-2.34933051	0.26622548	1.21199441
C	-2.75313935	0.83855483	0.00000000
C	-2.34933051	0.26622548	-1.21199441
H	-1.21886829	-1.31544210	-2.15224718
H	-2.66268300	0.71045748	-2.15426488
H	-3.38246949	1.72587316	0.00000000
H	-2.66268300	0.71045748	2.15426488
H	-1.21886829	-1.31544210	2.15224718
H	-0.49428144	-2.32421768	0.00000000
C	1.13568505	1.44656968	0.00000000
C	1.54059038	0.87585546	-1.21105772
C	1.54059038	0.87585546	1.21105772
C	2.34933051	-0.26622548	-1.21199441
C	2.75313935	-0.83855483	0.00000000
C	2.34933051	-0.26622548	1.21199441
H	0.49428144	2.32421768	0.00000000
H	1.21886829	1.31544210	2.15224718
H	2.66268300	-0.71045748	2.15426488
H	3.38246949	-1.72587316	0.00000000
H	2.66268300	-0.71045748	-2.15426488
H	1.21886829	1.31544210	-2.15224718

$(C_6H_6)_2$ (D_{6h}) [-3364.70]

C	0.00000000	1.39917723	-1.95673257
C	-1.21172302	0.69958861	-1.95673257
C	1.21172302	0.69958861	-1.95673257
C	-1.21172302	-0.69958861	-1.95673257
C	0.00000000	-1.39917723	-1.95673257
C	1.21172302	-0.69958861	-1.95673257
H	2.15370992	1.24344500	-1.95136270
H	2.15370992	-1.24344500	-1.95136270
H	0.00000000	-2.48689000	-1.95136270
H	-2.15370992	-1.24344500	-1.95136270
H	-2.15370992	1.24344500	-1.95136270
H	0.00000000	2.48689000	-1.95136270
C	0.00000000	-1.39917723	1.95673257
C	1.21172302	-0.69958861	1.95673257
C	-1.21172302	-0.69958861	1.95673257
C	1.21172302	0.69958861	1.95673257
C	0.00000000	1.39917723	1.95673257
C	-1.21172302	0.69958861	1.95673257
H	0.00000000	-2.48689000	1.95136270
H	-2.15370992	-1.24344500	1.95136270
H	-2.15370992	1.24344500	1.95136270
H	0.00000000	2.48689000	1.95136270
H	2.15370992	1.24344500	1.95136270
H	2.15370992	-1.24344500	1.95136270

Pyrazine stacked dimer (C_s) [-2933.66]

C	-1.27314787	-1.22864746	-0.69934393
C	-1.27314787	-1.22864746	0.69934393
N	-0.27833531	-1.78166580	1.41464629
C	0.71669589	-2.33067512	0.69968199
C	0.71669589	-2.33067512	-0.69968199
N	-0.27833531	-1.78166580	-1.41464629
H	-2.09022900	-0.77133430	-1.25513036
H	-2.09022900	-0.77133430	1.25513036
H	1.53791348	-2.78024444	1.25613678
H	1.53791348	-2.78024444	-1.25613678
C	-0.33336721	2.11960582	1.13730385
C	0.89001666	1.44076950	1.13735878
N	1.51616656	1.09572276	0.00000000
C	0.89001666	1.44076950	-1.13735878
C	-0.33336721	2.11960582	-1.13730385
N	-0.95930581	2.46656541	0.00000000
H	-0.82205239	2.38566863	2.07368561
H	1.37069276	1.16060526	2.07326367
H	1.37069276	1.16060526	-2.07326367
H	-0.82205239	2.38566863	-2.07368561

Indole•Benzene (C_s, T-shaped) [-4023.95]

C	2.51018656	1.66881359	0.00000000
C	2.72907686	1.00509185	-1.21293451
C	3.17029445	-0.32359612	-1.21322970
C	3.39170963	-0.98782818	0.00000000
C	3.17029445	-0.32359612	1.21322970
C	2.72907686	1.00509185	1.21293451
H	2.16535134	2.69963543	0.00000000
H	2.54947192	1.51817747	-2.15406212
H	3.33567357	-0.84105173	-2.15475096
H	3.73337517	-2.02006146	0.00000000
H	3.33567357	-0.84105173	2.15475096
H	2.54947192	1.51817747	2.15406212
H	0.78766070	-0.60255433	0.00000000
N	-0.18199375	-0.88951487	0.00000000
C	-0.62215588	-2.20429518	0.00000000
C	-1.99939458	-2.22819925	0.00000000
C	-2.44773480	-0.85946592	0.00000000
C	-1.27407139	-0.03910127	0.00000000
C	-1.34006867	1.36010688	0.00000000
C	-2.60479650	1.94724774	0.00000000
C	-3.77820041	1.15766885	0.00000000
C	-3.71021863	-0.23368872	0.00000000
H	0.08971879	-3.01743954	0.00000000
H	-2.61755626	-3.11577211	0.00000000
H	-0.43602019	1.96322010	0.00000000
H	-2.69086875	3.03087264	0.00000000
H	-4.74789224	1.64906015	0.00000000
H	-4.61911517	-0.83134401	0.00000000

Phenol Dimer (C_1) [-3663.30]

C	-2.05756688	0.74267869	-0.11656526
O	-1.37352081	1.88704231	-0.46985998
H	-0.49292290	1.89308629	-0.03980681
C	-1.52580652	-0.21790668	0.75852131
C	-2.26989099	-1.36058652	1.06925342
C	-3.54088019	-1.55609020	0.51797705
C	-4.06435309	-0.59160686	-0.35320491
C	-3.33103407	0.55315006	-0.67295730
H	-0.53659314	-0.07393326	1.18533315
H	-1.84827144	-2.10045177	1.74556546
H	-4.11524604	-2.44547911	0.76298067
H	-5.05121729	-0.73063411	-0.78826782
H	-3.72570554	1.30819411	-1.34783040
O	1.31180066	1.87446382	0.48268398
C	2.07507197	0.75074607	0.14887081
H	1.69078425	2.30172722	1.26937603
C	1.66203637	0.00496717	-0.95769245
C	2.39264920	-1.12903776	-1.31992578
C	3.52308863	-1.51530297	-0.58948357
C	3.92270043	-0.75784827	0.51633726
C	3.19863137	0.37911038	0.89253927
H	0.78020735	0.31007162	-1.51310828
H	2.07177973	-1.71388902	-2.17765626
H	4.08601535	-2.39837138	-0.87866479
H	4.79813012	-1.04730061	1.09161385
H	3.50594796	0.97095198	1.75409750
